# Hierarchical dynamics in allostery following ATP hydrolysis monitored by single molecule FRET measurements and MD simulations†

**DOI:** 10.1039/d0sc06134d

**Published:** 2021-01-15

**Authors:** Steffen Wolf, Benedikt Sohmen, Björn Hellenkamp, Johann Thurn, Gerhard Stock, Thorsten Hugel

**Affiliations:** Biomolecular Dynamics, Institute of Physics, University of Freiburg Freiburg Germany steffen.wolf@physik.uni-freiburg.de +49 761 203 5883 +49 761 203 5913; Institute of Physical Chemistry, University of Freiburg Freiburg Germany thorsten.hugel@physchem.uni-freiburg.de +49 761 203 6192; Engineering and Applied Sciences, Columbia University New York USA b.hellenkamp@columbia.edu; Signalling Research Centers BIOSS and CIBSS, University of Freiburg Freiburg Germany

## Abstract

We report on a study that combines advanced fluorescence methods with molecular dynamics (MD) simulations to cover timescales from nanoseconds to milliseconds for a large protein. This allows us to delineate how ATP hydrolysis in a protein causes allosteric changes at a distant protein binding site, using the chaperone Hsp90 as test system. The allosteric process occurs *via* hierarchical dynamics involving timescales from nano- to milliseconds and length scales from Ångstroms to several nanometers. We find that hydrolysis of one ATP is coupled to a conformational change of Arg380, which in turn passes structural information *via* the large M-domain α-helix to the whole protein. The resulting structural asymmetry in Hsp90 leads to the collapse of a central folding substrate binding site, causing the formation of a novel collapsed state (closed state B) that we characterise structurally. We presume that similar hierarchical mechanisms are fundamental for information transfer induced by ATP hydrolysis through many other proteins.

## Abstract 2

We report on a study that combines advanced fluorescence methods with molecular dynamics simulations to cover timescales from nanoseconds to milliseconds for a large protein, the chaperone Hsp90.

## Graphical abstract

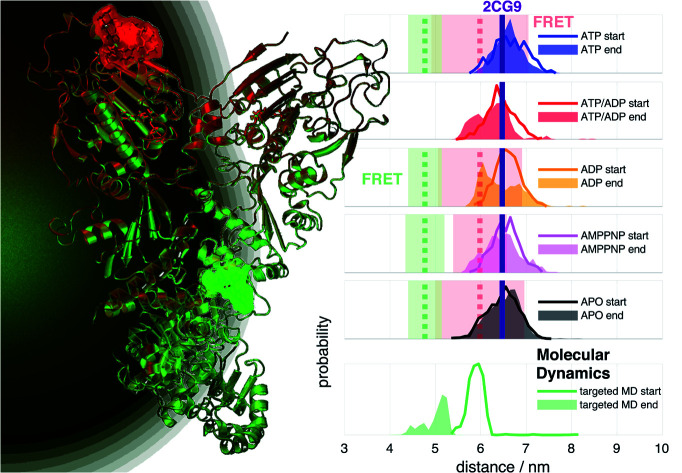

## Introduction

Allosteric communication, *i.e.*, coupling between distant regions of a protein, is an elementary mechanism for the regulation of protein function, signalling and energy transfer, as it enables alteration of protein structures at active sites by small changes in a binding pocket several nanometers away.^1,2^ In spite of its importance, there is surprisingly little known about the underlying dynamical process and the timescales of allosteric communication.^3–5^ Understanding the molecular mechanisms of allostery is crucial to, *e.g.*, elucidate the effects of point mutations in cancer development^6^ or to exploit it for the design of small molecule drugs.^7^

**Fig. 1:**
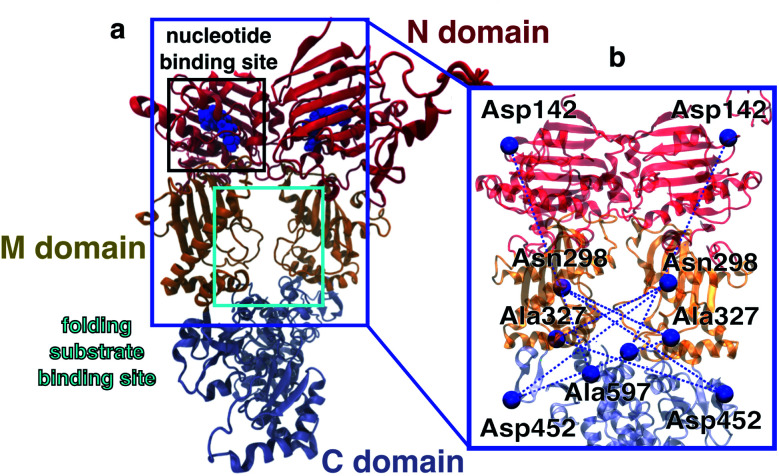
Overview of the Hsp90 dimer. (a) Initial protein model based on the X-ray crystal structure 2CG9.^8^ N-domain is shown in red, M-domain in orange, C-domain in light blue. Nucleotide bound to the N-domain as blue spheres. (b) FRET distance pairs used in this study.

In this work, we investigate allostery following ATP hydrolysis within the yeast molecular chaperone heat shock protein 90 (Hsp90). Hsp90 is highly conserved in eucariotic cells, is involved in many intracellular signalling pathways,^9,10^ serves, for example, as a cyclin-dependent kinase regulator, and is therefore a target protein for cancer treatment.^11^ The chaperone is a homodimer consisting of two copies from a 670 amino acid protein, with each of the chains containing three domains named N, M and C (see Fig. 1). The two monomers undergo large conformational changes, which are usually classified into N-terminal open and closed states.^8^ A nucleotide binding site is found within the N-domain (see Fig. 1a). Structural changes on the Ångstrom scale such as the hydrolysis of ATP into ADP are believed to cause significant conformational changes of the full dimer.^10,12^ Several X-ray crystallography and cryo-EM structures are available for the closed state.^8,13,14^ Structures of the open state have been proposed based on molecular dynamics (MD) simulations^15,16^ and by a combination of single molecule Förster resonance energy transfer (smFRET) and MD simulations.^17^ Despite this wealth of structural information, the molecular details on how nucleotides affect the dimer and how the nucleotide hydrolysis cycle is coupled to folding client binding and processing have remained elusive.^18^ Elucidating this connection could open the door for the rational design of novel small molecule Hsp90 inhibitors.

To investigate allosteric communication in Hsp90 across several time- and length scales, we carry out a joint experimental and theoretical study combining smFRET, fluorescence correlation spectroscopy (FCS) and fluorescence lifetime measurements with fully atomistic MD simulations. We use both intensity-based smFRET^19,20^ and single molecule lifetime and anisotropy measurements^21,22^ to determine distances between selected amino acid side chains in the M-domain of Hsp90 and investigate distance changes upon addition of different nucleotides. Complementary, we perform unbiased and biased all-atom MD simulations of the full dimer and loadings of different nucleotides (total simulation length of 45 μs) to obtain insights into molecular mechanisms which are not directly accessible with smFRET for such a large protein.^23,24^ We determine the accessible volume of the dyes^22,25,26^ for each structure from the MD simulations and calculate the expected mean distance between the two dyes, and believe that this is the best way to join smFRET measurements and MD simulations. We find that Arg380, which has been assumed to be involved in the functional cycle before,^27–29^ is a prominent residue for the transfer of allosteric information. Experimentally and computationally observed time- and length scales of allosteric communication agree well with each other, which allows us to propose a detailed molecular pathway from hydrolysis to a large conformational change of the M-domains.

## Results and discussion

### Intermolecular distance measurements

**Fig. 2:**
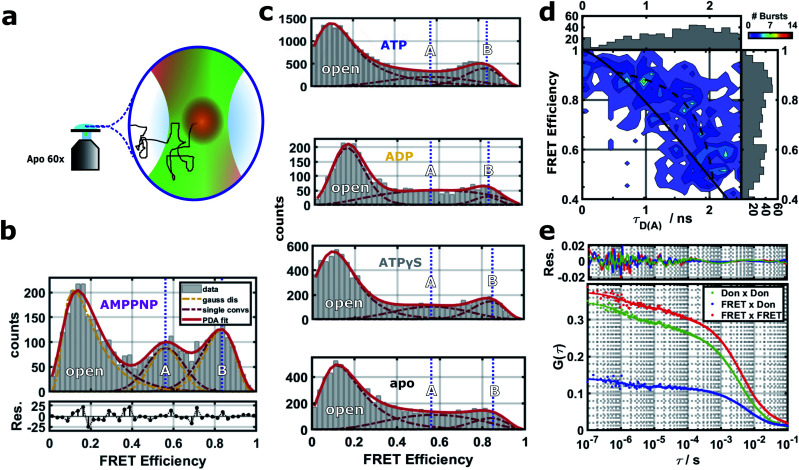
(a) Schematics of the confocal smFRET measurement. (b–e) Single-molecule FRET results for the distance 452–298 (see Fig. S3† for distances 298–452, 327–298, 327–452 and 142–597, and Fig. S4† for stoichiometry *vs.* FRET efficiency plots). (b) FRET efficiencies and their distributions are extracted by PDA,^30,31^ which includes a separation from shot-noise. The PDA fit (red line) results in three states (dark red dashed lines): an open state, a closed state A and a more contracted closed state B. For each population, mean distances *μ* were obtained from shot-noise filtered distributions (orange lines) as expectation values (vertical blue dashed lines). (c) FRET efficiencies under four different nucleotide conditions. Blue dashed lines correspond to the mean distances of the two closed states A and B, respectively. (d) FRET efficiency *vs.* donor lifetime *τ*_D(A)_ for the closed states of the Hsp90 variant 452–298 with AMPPNP. Theoretical static FRET line as straight black line, dynamic FRET line as dashed black line. (e) FRET-FCS analysis in the presence of AMPPNP. Correlations of the parallel and perpendicular donor signal (green), FRET signal (red) and the cross-correlation of the parallel FRET signal with the perpendicular donor signal (blue).

To describe the effect of nucleotides on the closed conformation of the Hsp90 dimer, we performed smFRET experiments in a home-built confocal microscope as described in detail before.^22^ We used pulsed interleaved excitation (PIE) with a green (532 nm) and a red (640 nm) laser. Parallel and perpendicular donor and acceptor fluorescence was detected by four single photon detectors and time-correlated single photon counting (TCSPC). Fig. 2a shows a schematic of a single-molecule measurement. We attached pairs of Atto550 and Atto647N fluorophores to the amino acid positions 298, 327 and 452 as well as the amino acids 142 and 597 (see the blue network in Fig. 1b). Three out of five of the labels are located in the middle domain of the protein dimer, *i.e.*, between M–M and M–C domains at a distance of 3.0–5.0 nm from the nucleotide binding site. 142–597 is a complementary N–C distance pair. Each FRET label pair was measured in the apo form and additionally in the presence of 2 mM ATP, ADP, AMPPNP or ATPγS (see ESI Methods and Fig. S1 and S2†). We note that while the protein contains ADP + P_i_ after hydrolysis, we observed no difference between ADP and ADP + P_i_ measurements (*cf.* Fig. S5†).

A Photon Distribution Analysis (PDA)^30,31^ of the corrected 2D FRET efficiency histograms Fig. 2b and c reveals three predominant distance populations for several of the measured protein/nucleotide complexes. These populations can be attributed to the existence of two closed protein structures (termed closed states A and B) in addition to the open protein conformation. The closed states can be best distinguished in the presence of AMPPNP where they are similarly populated. Therefore we used the mean FRET efficiencies of the three states from the AMPPNP data to constrain fits of other nucleotides. The width of the fits was not constrained as these three global states are dynamic and might show nucleotide dependent fine structure due to small structural differences (see below). The efficiency-averaged apparent distances between the dyes *R*_〈*E*〉_^22^ and their distributions are extracted by PDA for separation of distance distributions from shot-noise (see ESI Methods for details and Table S1 together with Fig. S3† for all measured distances).

The smFRET data in Fig. 2 shows quasi-equilibrium distributions. These distributions are used to define and distinguish the two closed states. The ratio of closed B to closed A is on average 0.23 ± 0.11 for apo, 0.65 ± 0.29 for ADP, 0.67 ± 0.21 for ATP, 1.21 ± 0.23 for ATPγS and 1.61 ± 0.34 for AMPPNP (error bars indicate the standard error of the mean). We interpret these results as follows: apo exhibits a equilibrium distribution of conformational states which dynamically interchange. ADP or ATP do not trap any of the closed A or closed B states, but only slightly modulate the dynamic equilibrium of nucleotide free Hsp90 towards closed state B. This is consistent with previous smFRET and bulk data.^12,32^ ATPγS (a slowly hydrolyzing ATP analog) and AMPPNP (an ATP analog that is not significantly hydrolyzed by Hsp90) cause Hsp90 to increase the population of some (closed) states, which is also consistent with the literature.^12,32^

Most interestingly, both nucleotide analogs (ATPγS and AMPPNP) seem to populate closed A and closed B, with a slight preference for closed state B. On first sight this is surprising, as the X-ray structure of Hsp90 with AMPPNP (PDB ID 2CG9)^8^ is only a single structure, from which we can calculate FRET distances that are similar to closed A state. This could be explained by a the following conformational selection mechanism: ATPγS and AMPPNP select closed A and closed B from all possible states of Hsp90 and increase their population. Then p23 and the mutations that were imposed to crystalize Hsp90 to obtain the crystal structure further select closed state A. Such a mechanism, in which the binding of ligands causes a partial shift in protein state population, but does not completely depopulate states, has been shown before for other systems.^5,33^ This mechanism would best explain our data, but has to be further investigated. A recent study on yeast Hsp90 also found two fast interconverting closed states.^34^ In contrast to our findings, the authors of this study identify the AMPPNP state as a prehydrolysis state. However, our pictures agree in that ATP hydrolysis results in a transition to an open state *via* a more compact ADP state.

### Timescales observed from lifetime and FRET-FCS data

Next we investigated on which timescales dynamics can be observed in our experimental data. First we tested for dynamics on the ms timescale by FRET efficiency *vs.* fluorescence lifetime plots.^35,36^Fig. 2d reveals signatures of dynamics on the ms timescale between the closed states A and B (see ESI Methods for more details†). A completely static sample should lay on the theoretical static FRET line. Lifetimes obtained from the reconvolution fit were used to determine start and end point of the dynamic FRET line, to which the data agree better. This is consistent with the analysis shown in Fig. 2b, because the additional population for the dynamic intermediate state between states A and B is only sparsely populated.

Then we performed a FRET-FCS analysis,^37,38^ useful for revealing potential dynamics occurring between 100 ns and 1 ms. A thorough analysis (see ESI Methods†) suggests correlation times on three timescales. The first correlation time on the order of a few milliseconds corresponds to the diffusion time of Hsp90. The two other correlation times most likely correspond to structural dynamics within the Hsp90 dimer. Fig. 2e displays a FRET-FCS analysis in the presence of AMPPNP. A global fit with two kinetic terms revealed dynamics on the timescale of about 1 μs and 60 μs, respectively, with a weight of around 10%, consistent with the lifetime-efficiency analysis. Performing FRET-FCS measurements with all other nucleotides revealed that the estimated timescales of the found dynamics are in the range of *τ*_L_ ≈ 50–200 μs and *τ*_K_ ≈ 1–4 μs and are shown in Fig. S6 and S7.†

Information on the structural dynamics are revealed by the correlative characteristics of the donor–donor correlations DonxDon, acceptor–acceptor correlations FRETxFRET and the FRET pair correlations DonxFRET, calculated based on TCSPC data. The correlation times at *τ*_L_ ≈ 50−200 μs contain a mostly anti-correlated DonxFRET signal which hints towards distance dynamics between the two dyes in the Hsp90 M-domain. At fast correlation times *τ*_K_ ≈ 1–4 μs we obtain correlated and anti-correlated signals superimposed. The correlated signal probably arises from changes in the donor accessible volume caused by dynamics between N- and M-domain (see ESI page 8, Fig. S6 and S7†). As the time scales do not change upon increasing the solvent viscosity (see Fig. S8†), we can exclude that they result from rotational diffusion.

### Structural changes of the Hsp90 dimer

To connect smFRET measurements and our MD simulations (see below), we calculated the expected distance distributions *P*(*R*_〈*E*〉_) for fluorophore pairs directly from intraprotein dye-accessible volumes of the simulated structures (see ESI Methods†). In this way, we can directly compare simulations with the measured mean distances and uncertainties.

**Fig. 3:**
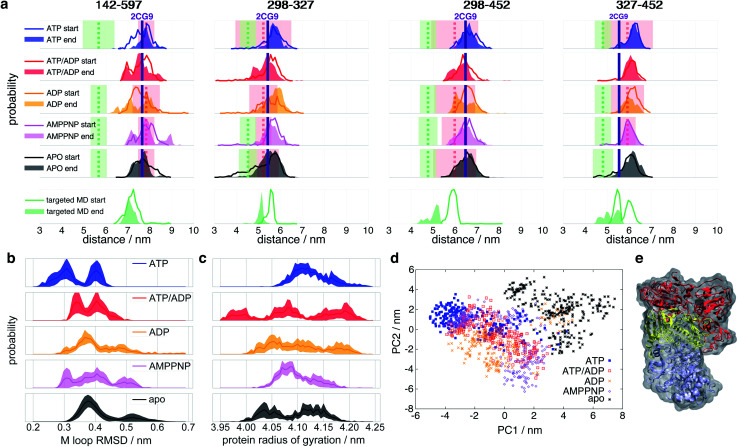
Histograms of selected Hsp90 distances. (a) Experimental and simulated apparent distances *R*_〈*E*〉_ of the FRET label pairs 298–327, 298–452, 327–452 and 142–597 (see ESI Methods for details†). Line histograms display the first 0.25 μs of simulation (“start”), filled histograms the last 0.25 μs (“end”). Vertical dashed lines indicate the measured mean distances (*R*_〈*E*〉_) of closed states A (red) and B (green), respectively, and coloured transparent areas the respective uncertainties from experiment (see Table S1†). Vertical purple lines indicate the 2CG9 structure state distance.^8^ Targeted MD started from an ADP state after 1 μs of unbiased MD. (b) RMSD of internal M-domain loop (sequence positions 323 to 340) during the last 0.25 μs with reference to positions in the active folding complex (PDB ID 5FWK).^14^ (c) Radius of gyration of the full protein during the last 0.25 μs of simulation. (d) Principal component (PC) analysis of the last 0.5 μs of simulations of intraprotein contact distances within nucleotide binding sites and M-loops. (e) Proposed model of closed state B based on nonequilibrium targeted MD simulations. Orientation and color code as in Fig. 1. Protein surface in grey.

Fig. 3a shows the means and uncertainties extracted from the experimental FRET efficiency histograms (see Fig. 2, S3 and ESI Methods for details†). State A (distances as red dashed line in Fig. 3a) occurs at distances comparable to the ones found within the crystal structure 2CG9,^8^ while state B (distances as green dashed line in Fig. 3a) represents a structure in which the protein is significantly contracted, and does not correspond to any previously structurally described state. At the start of the simulation we observe a qualitative agreement with experiment for distances attributed to the closed state A under all nucleotide conditions. In the case of ATP and AMPPNP, the simulations mainly remain in state A. However, in the presence of ATP/ADP and even more clearly in the presence of ADP, the distributions show some tendency towards closed state B. Missing convergence of MD simulations to state B indicates structural relaxation timescales longer than the maximal single trajectory length of 1 μs.

To enforce structural changes to closed state B and to overcome the timescale limitations of unbiased simulations, we employed nonequilibrium targeted MD simulations^39^ with implicit solvent for one of the ADP simulations. We pulled the C_β_ distances of amino acid pairs 298–327, 298–452 and 327–452 towards each other (see ESI Methods for details† and an explanation for the choice of these pairs). The targeted MD distance distributions indeed evolve towards the experimentally measured distances of the closed state B (see Fig. 3a, bottom). The *R*_〈*E*〉_ of these three pairs agree well with their experimental counterparts. The 142–597 distance pair, which we did not manipulate, also tends towards the experimental closed state B (green dashed line in Fig. 3a).

We observe that ATP-bound Hsp90 generally tends to narrow distance distributions and larger overall distances, indicating that closed state A is most stable with ATP (before hydrolysis) and opens a central hole between the monomers (see below). All other states tend to wider distributions and/or cover shorter distances that develop towards closed state B, *i.e.*, towards closing the hole.

Does this change in M–M distances have a functional consequence at other sites of the protein? To address this question, we compared our MD results to the Hsp90-Cdk4 cryo-EM structure (PDB ID 5FWK).^14^Fig. 1a shows that the folding substrate binding site is found directly in the core of the dimer between the two M-domains. The chain is held in place by two loops coming out of the M-domains (sequence positions 323–340 (ref. 14)). To compare the M-loop structures appearing during simulation to the corresponding cryo-EM structure, Fig. 3b displays the respective C_α_-RMSDs. The ATP-bound structure appears to switch between two conformations, one of which (RMSD ≈ 0.3 nm) is closest to the cryo-EM structure loop arrangement. Interestingly, AMPPNP resembles more the hydrolysed states than ATP. We further note that these M-loops are found at a distance of *ca.* 4.0 nm (quite far away) from the nucleotide binding site.

To assess structural differences of the full protein effected by different nucleotides, we also analyse the differences in the radius of gyration of the full dimer in Fig. 3c. The ATP-bound protein tends to a clearly defined large radius of gyration, while all other investigated states display a broader distribution and a trend to smaller radii. Again, AMPPNP states resemble more the hydrolysed states.

To obtain a model for the structural changes underlying *τ*_L_, we evaluate the data from our biased MD simulations (see ESI Methods for details†). The structural consequences are displayed in Fig. 3e: one of the monomers gains a kink at its M–C interfaces and bends over towards its partner monomer, resulting in an asymmetric contracted state, which we tentatively attribute to represent a structural model of the closed state B. This asymmetric state, in which one of the two monomers significantly differs in its form from its neighbor, has so far not been described.

### Nucleotide binding

**Fig. 4:**
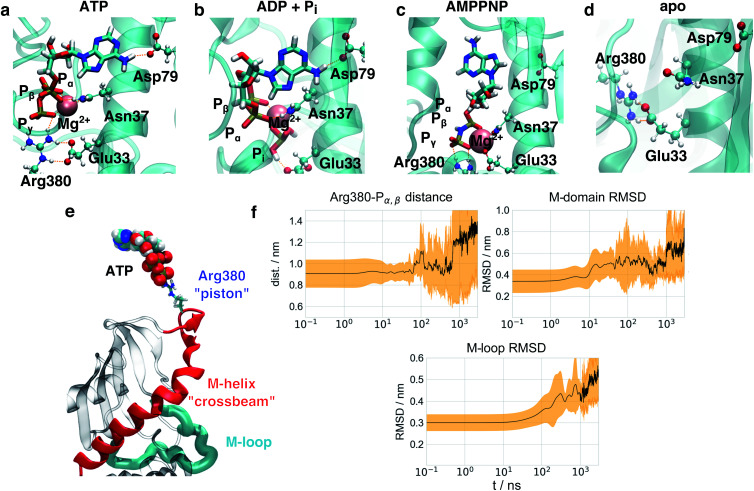
Transfer of structural changes from the nucleotide binding site to the full protein dimer. Nucleotide binding site in (a) ATP-bound, (b) ADP + P_i_-bound, (c) AMPPNP-bound and (d) apo-Hsp90 simulations after 1 μs. Nucleotides displayed as sticks, amino acid side chains as balls and sticks, protein backbone as cartoon, hydrogen bonds as orange dashes. In the case of ADP + P_i_, Arg380 has left the nucleotide binding site. (e) Essential mechanistic coupling of nucleotide binding site and the M-domain. (f) Time evolution of average structural changes observed in 3 μs ADP state simulations. Mean values displayed in black lines, standard deviations as yellow traces.

As a first step towards an understanding of the molecular basis of structural differences imposed by the nucleotides onto the protein, we focused on structural changes found directly at the nucleotide binding sites in simulations. Fig. 4a shows a representative structure of ATP bound after 1 μs simulation time. ATP is stably connected to the protein at Asp79 *via* the adenine moiety. As the loss in nucleotide affinity in the D79N mutant^40^ indicates, this residue and its negative charge are crucial for the correct anchoring of the adenine moiety. The P_γ_ phosphate forms a stable salt bridge with Arg380 which is in agreement with observations from NMR.^41^ Arg380 is highly conserved within the family of Hsp90 and its homologues^18,27^ and speculated to take part in allosteric information transfer.^28^ While the influence of Arg380 on ATP activity has been controversially discussed,^27,29,42,43^ Arg380 has indeed been considered as a potential residue to mediate the stabilisation of a specific N/M arrangement.^44^ It is not directly involved in the water deprotonation reaction,^45^ and a R380A mutant completely suppresses the formation of the closed state.^29,41^ Interestingly, a lysine residue Lys307 can be found in the MutL DNA mismatch repair protein^46^ as displayed in Fig. S11.† This protein belongs to the same protein superfamily as Hsp90 and exhibits a comparable overall protein architecture (albeit with a significantly shorter C-terminal domain). The respective K307A mutant exhibits a similar weak effect on ATPase activity as seen for Hsp90 R380A in ref. 44, hinting to a positively charged residue at this position to be essential for protein function, but not necessarily for ATPase activity. Arg380 is positioned directly at the tetrahedron surface of P_γ_ that needs to be accessed by an attacking water molecule. As can be seen in Fig. S11,† while the triphosphate is surrounded by well defied volumes with high probability of occupation *via* a water molecule,^47^ the guanidinium moiety effectively blocks water molecules from attacking P_γ_, explaining earlier experimental findings that ATP bound to Hsp90 exhibits a life time in the range of several seconds.^48^

In comparison to this arrangement, we observe significant deviations in the simulations with two ADP + P_i_ (see Fig. 4b), in the following abbreviated as ADP. The binding mode of the adenine moiety and the magnesium ion is unaffected. P_i_ interferes with the positioning of the Arg380 side chain and a salt bridge of Arg380 and Glu33. This particular glutamate has been shown to be involved in the ATPase activity of Hsp90, as well.^49^ In the apo form, the binding site exhibits a collapsed state. Glu33 and Arg380 again are found in a salt bridge, but the position of Arg380 is significantly different from the one in the nucleotide-bound states.

Interestingly, while ATP and AMPPNP simulations started from the same positions and Mg^2+^ coordination as given in the PDB ID 2CG9, the binding mode of AMPPNP (Fig. 4c) is significantly different from the one of ATP at the end of simulations. The binding mode of the adenine moiety of AMPPNP becomes unstable in four out of five simulations (see Table S2†), and the P_γ_–Arg380 salt bridge ruptures in one simulation. Furthermore, instead of the α, β, γ-coordination in ATP, only P_β_ and P_γ_ coordinate the magnesium ion. As a substitute, Glu33 becomes part of the coordination sphere of the magnesium ion, disrupting the Glu33–Arg380 salt bridge. This effect appears to be caused by the presence of a new hydrogen bond donor in form of the NH group between P_β_ and P_γ_ that forms contacts with surrounding polar residues, and changes in the electron distribution over the triphosphate.

To investigate the effect of the hydrolysis of only a single ATP molecule, we performed simulations with one ATP and one ADP + P_i_ bound, respectively (in the following abbreviated as ATP/ADP). In this case the binding mode of the adenine “anchor” residue of ADP is stable in three out of the five simulations (see Table S2†), while in the remaining simulations one or both anchors lose their contact with Asp79, respectively. A single hydrolysis event therefore already seems to destabilise nucleotide binding, which is consistent with our experimental results of Hsp90 heterodimers containing a non-hydrolizable E33A mutant on one monomer site (see Fig. S5† and below) and the observation that ATP hydrolysis is only required in one subunit of the Hsp90 dimer for correct function.^50^

### Allosteric communication between nucleotide binding site and the protein dimer

To identify the key amino acids responsible for the structural information transfer, we performed a correlation-based principal component analysis on the contacts^51^ made by amino acids forming the nucleotide binding site and the M-loop (see ESI Methods for details†). Fig. 3d displays trajectories of the various systems projected onto the resulting first two principal components. The principal components clearly separate apo- and ATP-states. AMPPNP, ATP/ADP and ADP partially cover the ATP- and apo-states, but also areas that neither correspond to the latter two states, which is in qualitative agreement with our results given above.

Investigating the importance of individual contacts on the first two principal components (Fig. S9†), we find that besides changes within the binding pocket and the M-loop, Arg380 is highlighted to exhibit pronounced changes as found above. Arg380 is indeed prominent with respect to allosteric communication, as it is the only residue which reaches into the nucleotide binding site, but is also part of the M-domain.

On the basis of this insight, Fig. 4e shows the key structural elements of a mechanistic model of allostery involving Arg380. Arg380 forms a salt bridge with the P_γ_ of ATP, and is positioned in a loop at the N-terminal end of a helix (sequence positions 376–408, in the following called M-helix), which is part of the M-domain. The helix runs across the face of the central β-sheet of the M-domain, and thus can apply tension and affect the position of the whole domain. The location of the M-domain position in turn affects the orientation of the M-loop and thus regulates the formation of the folding client binding site. In other words, Arg380 serves as a “piston” to connect ATP and the N-terminal end of the M-helix *via* electrostatic interactions. The M-helix in turn represents a “crossbeam” that exerts force on the M-domain and effects the formation of the folding client binding site.

It is instructive to consider the effect of this mechanistic coupling on the different nucleotide states (Fig. S10†). In the ATP-bound state, we observe that the Arg380–P_γ_ salt bridge is present in both monomers. Arg380 pulls on the M-helices in both monomers, resulting in a very symmetric dimer structure. In the case of ATP/ADP, on the other hand, the free phosphate stays within the binding site and disturbs the position of one of the two Arg380 side chains. In this case, Arg380 moves away from the nucleotide. The Arg380 side chain in the other monomer with ATP still pulls in its M-helix. The resulting imbalance in connectivity between both monomers introduces a structural asymmetry, which has been observed to exist in other experiments.^52^ In the case of full hydrolysis (ADP), the retraction of Arg380 from the nucleotide binding site is present for both monomers, likely recovering structural symmetry.

We emphasise that the structural symmetry of the protein seen here is not effecting a symmetry in ATP hydrolysis. ATP hydrolysis appears to be a stochastic event, leading to the ATP/ADP state as the most probable post-hydrolysis structural intermediate. Hydrolysis appears to happen independently in both monomers, and thus a single hydrolysis event is the most likely one. To support this assumption, we have compared the symmetric dimer E33A–E33A with the asymmetric dimer E33A–WT. The E33A mutant is known to retain ATP affinity, but to inhibit hydrolysis.^40,42,49^ As can be seen in Fig. S5,† the double-mutant dimer enriches the closed state in the presence of ATP, while the asymmetric dimer is mainly found in the open state. Therefore, the large conformational changes of Hsp90 (*i.e.* the N-terminal opening) can already be induced by a single hydrolysis event. As in the case of the wild type Hsp90 in presence of AMPPNP or ATPγS, we observe the stabilization of both closed states A and B in E33A–E33A, further strengthening our hypothesis that the protein exhibits dynamics between both states in equilibrium.

It is interesting to note that in the proposed mechanism, the major role of ATP is the addition of electrostatic interactions to the chaperone, stabilising a client-binding competent conformation *via* induced structural stress. This hypothesis is in line with the observation that ATP exhibits a comparatively long life time in the binding pocket.^48^ Hydrolysis then returns the chaperone into an inactive state with a wide range of accessible conformations and, from a thermodynamic perspective, completes the overall energetic balance for the folding process. We therefore hypothesise that ATP acts as a regulatory element activating Hsp90 instead of directly performing work on the folding client. The main driving force of the presented mechanism is not to create new residue interaction networks, but to release structural tension from the Arg380–ATP electrostatic interaction.

### Timescales of structural changes in MD simulations

The model of allosteric communication in Hsp90 developed above suggests a sequence of information flow from the nucleotide binding site to the folding client binding site. To test this idea, we perform a time series analysis of our trajectories. Based on our findings on the nucleotide binding site, the simulation starting structure based on AMPPNP-bound 2CG9 is a mixture between an ATP-like and a partially hydrolysed state. We therefore consider the five ADP and ATP/ADP trajectories, respectively, as equilibration simulations from a non-equilibrium starting structure after hydrolysis, and extended them to a total of 5 × 3 μs. To monitor intra-protein changes associated with this allosteric communication, we focus on (i) the distance *d*_ArgP_ between the Arg380 side chain guanidinium carbon atom and the P_α,β_ mass center, (ii) the C_α_-RMSD of M-domain position (using C_α_-atoms of the N-domain as fit reference) and (iii) the C_α_-RMSD of the M-loop arrangement (residues 323–340) in reference to the cryo-EM structure 5FWK. *d*_ArgP_ highlights changes within the binding pocket directly after hydrolysis at a distance of ∼0.4 nm from the triphosphate, the M-domain RMSD protein report on conformational changes at intermediate (∼2.0 nm) distance, and the M-loop RMSD reflects conformational changes at the folding client binding site (∼4.0 nm distance).

Fig. 4f and S10† display the time evolution of means and standard deviations of these observables for ADP, ATP/ADP and ATP, averaging over 5 trajectories in each case. A complementary visualisation of changes in ADP simulations is given in ESI Movie 1.† The first differences appear directly at the start of simulations at *t* ≲ 1 ns: in ATP simulations, *d*_ArgP_ is constant and exhibits only small fluctuations at all times. The simulations with ADP start with an increased *d*_ArgP_ (∼0.15 nm longer) and exhibit larger fluctuations, reflecting a motion of Arg380 away from the nucleotide caused by the mobile free phosphate. These differences appear directly in the initial structure minimisation. In experiment, this conformational change is expected to occur following hydrolysis on a sub-ns timescale. While the initial P_γ_–Arg380 and Glu33–Arg380 salt bridges are well-defined in the ATP state, we could not find any specific contact pattern for these two amino acids in the trajectories after hydrolysis, which is reflected in the large standard deviation of ADP–Arg380 distances in Fig. 4. It appears that the ATP-bound protein represents an ordered state, which becomes disordered (and thus exhibits an increased entropy) after hydrolysis. In a similar vein, we do not observe any defined changes in the binding partners of Glu33.

The detachment of Arg380 from the nucleotide is a prerequisite for the subsequent rearrangements of the M-domain, which occur on a 10 ns timescale. In fact, at *t* ≳ 10 ns, the M-domain RMSD in the ADP system abruptly rises to *ca.* 0.5 nm. This change corresponds to an increased flexibility of the M-helix, followed by a rotation of the M-domains (see ESI Movie 1†). For ATP/ADP (see Fig. S10d†), we find a similar increase in M-domain RMSD, which appears to be slowed down and completes after 100 ns, pointing to a residual stabilisation of the active folding conformation from the single bound ATP. We mention that also in the ATP simulations an initial increase in M-domain RMSD to ∼0.4 nm takes place at similar times, followed by a delayed jump to a value similar to the one in the ADP simulations at *ca.* 300 ns. We interpret this change to come from the ATP simulations undergoing structural changes towards the folding-competent state. This involves conformational changes that are different from the one in the ADP simulations, but apparently follow a similar sequence of events.

The rotation of the M-domains is a prerequisite for the collapse of the folding client binding site, which is indicated by the M-loop RMSD and appears for *t* ≳ 100 ns. While the mean RMSD increases to *ca.* 0.35 nm in ATP, it reaches *ca.* 0.45 nm in ADP, which is in agreement with the distance-resolved RMSD profile in Fig. 3b. Lastly, *d*_ArgP_ strongly and continuously increases after *t* = 0.8 μs in ADP and exhibits large fluctuations, representing a complete detachment of Arg380 from the nucleotide binding site. This increase is not observed for ATP/ADP, again pointing to a remaining structural stabilization in case of a single hydrolysis event. Concomitantly, the M-domain RMSD jumps to 0.7 nm after 1 μs  and the M-loop RMSD continuously rises after 1 μs in ADP. In agreement with these findings from the MD simulations we see a fast timescale of *τ*_K_ ∼ 1 μs  in the FRET-FCS experiments. In ATP/ADP, the M-domain RMSD remains at ∼0.55 nm, and the M-domain RMSD at ∼0.4 nm, not displaying the increases observed for ADP.

The observed process timescales apparently follow a logarithmic scale, which is a hallmark of hierarchical dynamics, where fast processes associated with small free energy barriers regulate slow transitions:^54^ changes of the Arg380 position within ≲1 ns cause M-domain rearrangements on a 10 ns scale, which in turn are followed by sub-μs conformational changes of the M-loops forming the folding client binding site. The missing convergence to state B-associated distances implies that the full transfer from state A to the proposed state B takes even longer. In FRET-FCS we observe an additional timescale of *τ*_L_ of around 100 μs and in our lifetime analysis partially slower dynamics. As we already find a tendency towards state B in the longest MD simulations, we attribute this range of dynamics to transitions from state A to state B.

We note in passing that the hierarchical time scales from the MD simulations represent steady state (constant turnover) rates from state A towards state B, while the time scales from our FRET-FCS experiments result from an equilibrium between states A and B for all nucleotide conditions. Accordingly, we think that the observed timescales have the same molecular origin. This is in agreement with Onsager's regression hypothesis,^55^ namely that the equilibration of a non-equilibrium system (hierarchical progression from state A to state B) follows the same mechanism or path as equilibrium fluctuations.

## Conclusions

**Fig. 5:**
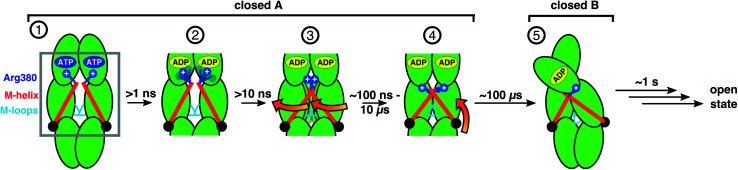
Scheme of allosteric communication including timescales. Steps 1–4 can be observed in unbiased MD simulations, steps 4 and 5 are in agreement with smFRET and FRET-FCS data and targeted MD simulations. Opening of the dimer has been investigated before and occurs on the timescale of seconds.^53^ Note that our data indicate that the hydrolysis of one ATP is already sufficient to induce the structural changes towards closed state B (see Fig. S10†).

We are now in a position to formulate a model of allosteric communication involving Arg380 and the M-helix depicted as steps (1)–(5) in Fig. 5: starting with ATP (1), Arg380 forms a salt bridge with P_γ_, and the resulting electrostatic interaction is transmitted *via* the M-domain to the M-loops, keeping the folding substrate binding site open. In the following steps, sub-ns hydrolysis cleaves the connection between nucleotide and piston (2), and within about 10 ns the strain exerted by the M helix on the M-domains is lost and the protein relaxes into an entropically favorable distribution of conformations (3) that form contracted states on a timescale of several hundred nanoseconds, where the M-domains are closer to each other and have rotated (4). All these four steps occur within state A. Finally, on the timescale of several hundreds of μs the dimer contracts into the asymmetric state B (5). Such hierarchical timescales for the individual steps in this allosteric communication hint towards a rugged free energy landscape involving several tiers underlying transitions.^4,54^

For the design of chemotherapeutics, novel inhibitors of Hsp90 could thus be based on creating small organic molecules that occupy the Arg380 position in the N-domain or interfere with the Glu33–Arg380 salt bridge or that pull on Arg380, similar as to the action of ATP, thereby forcing the protein into a long-lived extended state and preventing it from getting into the contracted state. While the presented channel of allosteric communication transfers structural information from the nucleotide to the M-domains, we note that it only forms a small part of possible allosteric interactions within the dimer, which can be mediated *e.g.* by co-chaperones^8^ or folding clients.^14^

The presented combination of smFRET, FCS and lifetime measurements with extensive nonequilibrium MD simulations holds the promise to investigate time-resolved molecular mechanisms of signalling and regulation in many other biomolecular systems. Last but not least, we believe that our findings are not only key to a better understanding of the Hsp90 machinery, but will contribute to generally recognise the allosteric information transfer through proteins.

## Experimental

### smFRET measurements

Single molecule measurements were carried out on an home-build confocal microscope as depicted in Fig. 2a. FRET efficiency and apparent donor–acceptor distance *R*_〈*E*〉_ between the dye-pairs were determined as in ref. 22. FRET-FCS and lifetime data were evaluated with the PAM software.^56^ Details on biochemistry and sample preparation, uncertainties in single-molecule experiments and the detailed analysis of the experiments are described in the ESI Methods, Fig. S1 and S2.†

### MD simulations and data analysis

The yeast wild type Hsp90 dimer model was based on the yeast Hsp90 crystal structure (PDB ID 2CG9).^8^ All simulations of the Hsp90 dimer water were carried out using Gromacs 2016 (ref. 57) using the Amber99SB*ILDN-parmbsc0-_χOL3_ + AMBER99 ATP/ADP force field.^58^ Details on protein modeling and the force field, the parameter generation procedure, unbiased simulations and nonequilibrium targeted molecular dynamics simulations,^39^ correlation-based contact principal component analysis^51^ and comparing smFRET data to MD simulation data are given in the ESI Methods.

## Conflicts of interest

There are no conflicts to declare.

## Supplementary Material

SC-012-D0SC06134D-s001

SC-012-D0SC06134D-s002
