# Supplementary material for: Hierarchical dynamics in allostery following ATP hydrolysis monitored by single molecule FRET measurements and MD simulations
Source: Chem Sci. 2021 Jan 15;12(9):3350–9. doi: 10.1039/d0sc06134d (PMC8179424; doi:10.1039/d0sc06134d)
Supplement: SC-012-D0SC06134D-s002 [file SC-012-D0SC06134D-s002.pdf]

Electronic Supplementary Material (ESI) for Chemical Science.
This journal is © The Royal Society of Chemistry 2021

# Supplementary Information: Hierarchical dynamics in allostery following ATP hydrolysis monitored by single molecule FRET measurements and MD simulations

Steffen Wolf*†,[1] Benedikt Sohmen†,[2] Björn Hellenkamp*,[2,3] Johann Thurn,[2] Gerhard Stock,[1] and Thorsten Hugel*[2,4]

[1)] *Biomolecular Dynamics, Institute of Physics, University of Freiburg, Freiburg, Germany*

[2)] *Institute of Physical Chemistry, University of Freiburg, Freiburg, Germany*

[3)] *Engineering and Applied Sciences, Columbia University, New York, USA*

[4)] *Signalling research centers BIOSS and CIBSS, University of Freiburg, Freiburg, Germany*

## SUPPLEMENTARY METHODS

### Biochemistry and sample preparation.

Yeast Hsp90 was recombinantly produced in *E. coli* and purified as described before.[1] Point mutations were inserted into the yeast Hsp90 gene to obtain single cysteines variants. Fluorescent labels were site-specifically introduced by cysteine-maleimide chemistry. We used ATTO550 as donor and ATTO647N as acceptor fluorophore, respectively (ATTO-TEC, Siegen, Germany). An inserted coiled-coil motif (DmKHC, *D. melanogaster*) at the C-terminus of Hsp90 prevented dimer dissociation and therefore increased the local concentration as we have shown before.[2] To obtain heterodimers which contain only one donor and one acceptor fluorophore, homodimers with the respective dye were mixed in an 1:1 ratio and incubated for 40 min at 43°C. This enabled a monomer-monomer exchange stochastically leading to 1/2 heterodimers and 1/2 homodimers. When the cysteine positions were different for donor and acceptor (e.g. for FRET pair 298-452) we performed dye-swapping experiments: for the first measurement, we labeled homodimers having the cysteines at positions X with donor (here 298) and homodimers having the cysteines at position Y with acceptor (here 452) before mixing. For the second measurement we labeled homodimers having the cysteines at positions X with acceptor and homodimers having the cysteines at position Y with donor before mixing. The subsequent monomer-exchange enabled to measure heterodimers with donor and acceptor positions swapped in two independent experiments. To remove aggregates, samples were centrifuged for 1h at 4°C and 16900 g. We applied size exclusion chromatography (SEC) to check that the amount of possibly remaining aggregates is sufficiently low (see Fig. S1 for details). The biofunctionality of the new variant was tested by ATPase assays (see Fig. S1).

### smFRET measurements.

Single molecule measurements were carried out on a home-build confocal microscope as depicted in Fig. 2a. Pulsed green and red laser light (532nm, LDH-P-FA-530 and 640nm, LDH-D-C-640, respectively, PicoQuant) was polarised, overlaid and focused on the sample by an 60x water immersion objective (CFI Plan Apo VC 60XC/1.2 WI, Nikon). Excitation light was separated from the emitted light by a dichroic mirror (F53-534 Dual Line beam

splitter z 532/633, AHF). The emitted light was then guided through a further dichroic mirror (F33-647 beam splitter 640 DCXR, AHF) to separate donor and acceptor fluorescence. After spectral separation pinholes with a diameter of $150\mu$m refined the detection volume to 8fL. Finally, the two photon streams were separated by polarizing beam splitters into their parallel and perpendicular parts and recorded by single-photon detectors (two SPCM-AQR-14, PerkinElmer and two PDMseries APDs, Micro Photon Devices). Time-correlated single photon counting with picosecond resolution and data collection was performed by a HydraHarp400 (PicoQuant) and the Symphotime 32 software (PicoQuant). To reach the single-molecule level we adjusted the protein concentration to about 50 pM. Measurements were recorded for 1800s. All experiments were carried out at 22°C in 40 mM HEPES, 150 mM KCl and 10mM $MgCl_2$ at pH=7.5. ADP and ATP were added to the protein solution directly before the measurement, in case of AMPPNP and ATP$\gamma$S, the samples were pre-incubated for 30 minutes. Nucleotides were added such that their final concentration was 2 mM.

**smFRET data analysis.**

Donor and acceptor photon streams were collected into 1 ms bins. Bursts were selected by a minimum threshold of 100 photons. For every burst the FRET efficiency $E$ and a stoichiometry $S$ was determined as detailed in Ref.[3]. FRET populations were determined within the region of $0.3 < S < 0.7$ of corrected $E$ vs $S$ plots. From the FRET efficiency an apparent donor-acceptor distance $R_{\langle E\rangle}$ between the two dyes is determined as[3]

$$R_{\langle E\rangle} = R_0 \left(E^{-1} - 1\right)^{\frac{1}{6}} , \tag{1}$$

where $R_0$ denotes the Förster radius. We use the efficiency-averaged apparent distance $R_{\langle E\rangle}$, because we assume that during a burst the complete accessible volume of the respective dye is sampled homogeneously, i.e., $E$ is already an average efficiency. $R_{\langle E\rangle}$ can be calculated by the FPS software[4] as is visualized in Fig. S2. Note that $R_{\langle E\rangle}$ is also used as a variable for the average from many efficiency averaged bursts, which is only equivalent to $R_{\langle E\rangle}$ in Equation 1 if protein dynamics is negligible.

For every FRET label pair, the Förster radius $R_0$ was calculated from the donor quantum

efficiency $Q_\mathrm{D}$, the spectral overlap $J$, and the relative dipole orientation factor $\kappa^2$

$$R_0^6 \propto \kappa^2\, Q_\mathrm{D}\, J. \tag{2}$$

Here the dipole orientation factor is assumed to be $\langle\kappa^2\rangle = 2/3$ for isotropic coupling. The position-specific donor quantum yield $Q_\mathrm{D}$ is given in terms of in dependence of the measured lifetime $\tau_\mathrm{D}$ as well as the parameters $Q_\mathrm{D0}$ and lifetime $\tau_\mathrm{D0}$ specified by the manufacturer[5] as

$$Q_\mathrm{D} = Q_\mathrm{D0}\frac{\tau_\mathrm{D}}{\tau_\mathrm{D0}}. \tag{3}$$

Based on the efficiency distributions (see Fig. 2, S3 and S4) and the respective $R_{\langle E\rangle}$ distribution, we determined the expectation value $\mu$ of the distance between the two dyes and the apparent distance fluctuation $\sigma$ via a Photon Distribution Analysis (PDA).[6,7] To that end, we fitted a shot-noise-broadened sum of Gaussian distance distributions to our $E$ histogram, and performed an axis transformation from $E$-space to $R_{\langle E\rangle}$-space via Equation (1). Specifically, we transform only the bin edges. The Gaussian distance fraction of each histogram bin is obtained by integrating from the left to the right edge and extracting the mean distance. Additionally, the $E$-specific shot-noise contribution is calculated for each bin, which can be described by a beta-function. We derive its parameters from the number of photons per burst, direct excitation and leakage similar to Ref.[7]. The final convolution is gained by simply summing all bin-wise shot-noise beta-functions weighted with the value of the Gaussians for each bin. This essentially assumes a distance delta-function (no protein dynamics) for each bin that undergoes a broadening by shot-noise, which simplifies the convolution greatly. The amplitudes, mean values $\mu$ and width $\sigma_\mathrm{Gauss}$ of each Gaussian are then optimised by minimising the squared residuals between data and fit.

### Uncertainties in single-molecule experiments.

The widths of the shot-noise-broadened Gaussian fits is caused by shot-noise and structural fluctuations of the protein itself. While $\sigma_\mathrm{Gauss}$ is therefore not directly related to an uncertainty of the mean value $\mu$, in our case it is a good indication for the uncertainty of the fit. In addition, in Fig. 4 we indicate a minimum uncertainty of 0.36 nm based on findings from structural remodelling.[5]

**Lifetime analysis.**

To investigate dynamics of the Hsp90 closed state on the millisecond timescale we performed FRET efficiency $E$ vs donor-lifetime-in-the-presence-of-acceptor $\tau_{D(A)}$ analysis using the PAM software[8] according to Refs.[4,9,10]. Herein, kinetics can be identified by comparing the experimental burst distribution to theoretical lines: the 'static FRET line'for a completely static sample and the 'dynamic FRET line'. To obtain the endpoints of the dynamic FRET line we applied sub-ensemble lifetime analysis. Using the filters $0.3 < S < 0.7$ and $0.65 < E < 1.0$ we obtained all bursts and microtimes belonging to closed state B. The microtimes from the parallel and perpendicular donor channels after green excitation were histogrammed. The instrument response function (IRF) was obtained from a pure water measurement. Correct channel alignment was achieved by correcting for IRF shifts due to count rate dependent timing. Different detection efficiencies for parallel and perpendicular detection were considered by the G-factor (see the end of the paragraph for a summary of correction parameters). A reconvolution fit with a bi-exponential model function was applied to the fluorescence decay and provided the dynamic FRET line endpoints. Note, that we did not use constraints for the lifetimes which could explain the slight deviations between the FRET efficiency of closed state B here (0.9) and the $ES$ plot in Fig. 2a.

We determined the lifetime fitting errors in two ways: first we calculate the lifetime standard deviation for four independent measurements of 298/452 with AMPPNP, which results in standard deviations of 0.06 ns and 0.16 ns for $\tau_1$ and $\tau_2$, respectively.Second, we determined the fitting error for the data set shown in Figs. 2d and S2 by parameter variation. $\chi^2_{\mathrm{red}}$ values were calculated by varying one lifetime while all other fit parameters were fixed to their fit minimum. For $\tau_1$ we obtained a range of 13% in which $\chi^2_{\mathrm{red}}$ did not change more than 0.01. For $\tau_2$ we obtained 7%. These values agree well with the standard deviations obtained in the first case.

$E$ vs. $\tau_{D(A)}$ analysis was performed for the stoichiometry-filtered burst subset of the Hsp90 closed state region. The bursts are smeared along the dynamic FRET line which is evidence for millisecond dynamics of Hsp90 closed state A and B. We emphasise that the dynamic line is not the result of a fit, but an analytic solution.[9–11] Comparing the measured data to these limiting cases, Fig. 2d shows that the data clearly fits better to the dynamic line. Please note that the dynamic intermediate state between states A and B is only very

little populated and therefore not defined as a state in the PDA analysis of Fig. 2.

Summary of correction parameters for the lifetime fit of Hsp90 closed state B:

| Start | Length | IRF Length | IRF Shift | Scat Shift | Perp Shift | G-factor |
|---|---|---|---|---|---|---|
| 8 | 322 | 71 | 5.2 | 4.9 | -1.7 | 1.2 |

Note, that for consistence we use the same correction parameters as they are declared in the PAM software[8]. ‘Start’ denotes start channel of the data with respect to the PIE channel start, ‘Length’ is the data range, ‘IRF length’ determines the channels which are used for IRF reconvolution, ‘IRF Shift’ the IRF shift with respect to the sample fluorescence, ‘Scat Shift’ the shift of the scatter pattern with respect to the sample fluorescence, ‘Perp Shift’ accounts for differences of the IRF shift in the parallel and perpendicular channel and the G-factor considers differences of parallel and perpendicular detection efficiencies.

**FRET-FCS analysis**

FRET-FCS measurements were carried out in SiPEG surface-passivated chambers at 22°C. Protein concentrations were in the range of 500 pM to 1.5 nM and the buffer used was 40 mM Hepes, 150 mM KCl and 10 mM $MgCl_2$ at pH=7.5. Measurement times were 600s and measurements were repeated at least once. By combining time-correlated single-photon counting (TCSPC) with channel information we link photon arrival times to donor and acceptor excitation. A DonxDon correlation results from the reemission of a photon from the donor molecule, a FRETxFRET correlation results from reemission of FRET-sensitized acceptor molecules and DonxFRET cross-correlation from their interaction. According to Barth et al.[12] we calculated DonxDon, FRETxFRET and DonxFRET using the PAM software[8] and Matlab R2017a. We tested several fit models, also including triplet terms (see below of an overview of the tested models). In a first approach we fixed the triplet fraction and triplet relaxation time to values that were separately determined to reduce the number of free parameters. We measured free Atto550 and free Atto647N dyes in solution and applied fit model A which contains a simple 3D diffusion term and a triplet term. However, as triplet characteristics are sensitive towards dye environments and further experimental parameters (e.g. laser excitation power), it is essentially preferable to derive them from the experiments directly. Therefore, we additionally calculated acceptor acceptor correlations

(par x perp) for each data set. Obtained triplet times $\tau_T$ were in the range of 1-10$\mu s$ with fractions around 5% which is consistent with previous studies[13]. This was also expected because we used sufficiently low laser powers which were within the linear range of a laser power vs. count rate plot (not shown here).

As the triplet fraction is very low, we proceeded similar to Barth et al.[12] and we fit the experimental data without a triplet term but with a kinetic term instead (model B). We obtained a fast kinetic rate which is on the same timescale as the triplet rate but with a fraction of more than 10%. We concluded, that an additional process on this timescale must be present which is not related to the triplet state of the dyes used. Finally, best fits were obtained in model C, which includes two kinetic terms and no triplet term. Thereby we obtained a second slower kinetic rate on the hundred microsecond timescale. Note, that the fact, that we can fit DonxDon, FRETxFRET and DonxFRET with model C whereas AccxAcc is well described by model A, is a negative control, because for AccxAcc one would not expect to see conformational dynamics.

Based on the idea of separating conformational dynamics from diffusive dynamics[11] we fit DonxDon, FRETxFRET and DonxFRET with globally linked relaxation times $\tau_K$ and $\tau_L$. We obtained diffusion times $\tau_D$ in the range of 2-4 ms, depending on the analysed FRET pair and the nucleotide condition. As aggregates impede correct interpretation of correlation data we applied the software-integrated algorithm for filtering[8]. To stay consistent we applied the same aggregate filter parameters for all data sets (threshold 40, time window 10, add window 3).

**Tested fit models:**

Model A

$$G(\tau) = \frac{\gamma}{N}(1 + \frac{T}{1-T}e^{-\frac{\tau}{\tau_T}})(1 + \frac{\tau}{\tau_D})^{-1}(1 + \frac{\tau}{\rho^2\tau_D})^{-0.5} + \text{const.}$$

Model B

$$G(\tau) = \frac{\gamma}{N}(1 + Ke^{-\frac{\tau}{\tau_K}})(1 + \frac{\tau}{\tau_D})^{-1}(1 + \frac{\tau}{\rho^2\tau_D})^{-0.5} + \text{const.}$$

Model C

$$G(\tau) = \frac{\gamma}{N}(1 + Ke^{-\frac{\tau}{\tau_K}} + Le^{-\frac{\tau}{\tau_L}})(1 + \frac{\tau}{\tau_D})^{-1}(1 + \frac{\tau}{\rho^2\tau_D})^{-0.5} + \text{const.}$$

Assuming a gaussian shape of the confocal volume we set the geometric factor $\gamma$ to $1/\sqrt{8}$. $N$ is the average number of particles in the confocal volume. $T$ and $\tau_T$ are triplet fraction and triplet relaxation time, respectively. Diffusion is characterized by the diffusion time $\tau_D$. $\tau_K$, $\tau_L$ with fractions $K$ and $L$ describe two relaxation times with corresponding fractions, respectively. The setup-related factor $\rho$ describes the ratio between the axial and lateral diameter of the confocal volume and was determined beforehand. It was measured before each experiment by scanning of matrix-immobilized polymer beads which are smaller than the diffraction limit. Values for green and red varied from 3.4 to 4.1. Furthermore, a constant was included to account for dynamics occurring at timescales exceeding the experimental observation time.

Our FRET-FCS curves show multiple effects superimposed. The feature of a simultaneous increase of FRETxFRET and DonxDon signals with a decrease in the DonxFRET signal can be attributed to anti-correlated dynamics, which are FRET-related distance dynamics. Correlated dynamics are characterized by a simultaneous increase in all correlations (DonxDon, FRETxFRET and DonxFRET). They can be caused by rotational diffusion of the proteins or by side chain movements or by relative domain motions. Both, side chain movements and relative domain motions can result in temporal changes of the dye accessible volumes which in turn can change the average dipole orientation or the local gamma factor of the dyes. A change in the donor accessible volume results in a correlated signal. We cannot rule out contributions from protein rotational diffusion, but we believe that these are less likely, because in this case we would expect a larger weight of the fast dynamics in the acceptor-acceptor correlation. The contribution due to leakage is negligible, because it affects all timescales similarly.

### Anisotropy criterion

Sufficient rotational freedom of protein-coupled FRET dyes is a prerequisite for reliable distance measurements and can be measured by time-resolved anisotropy experiments[5]. Nucleotide- and subpopulation-specific time-resolved anisotropies were determined for each FRET pair. We did so by identifying all photons of a population from the 2D $ES$ plot, histogramming their microtimes and calculating the anisotropies $r(t)$:

$$r_{\mathrm{DD}}(t) = \frac{I_{DD}^{\parallel} - g_G I_{DD}^{\perp}}{I_{DD}^{\parallel} + 2 g_G I_{DD}^{\perp}} \tag{4a}$$

$$r_{\mathrm{AA}}(t) = \frac{I_{AA}^{\parallel} - g_G I_{AA}^{\perp}}{I_{AA}^{\parallel} + 2 g_R I_{AA}^{\perp}} \tag{4b}$$

Here, the subscripts DD and AA denote photons from the green channel after green excitation and photons from the red channel after red excitation, respectively. Polarisation of the photons is indicated by the superscripts $\parallel$ and $\perp$. $I$ is the number of photons in the respective microtime bin and $g$ a detection correction parameter for green ($g_G$) and red ($g_R$) detection. The combined residual anisotropies were calculated as a geometric mean of the donor and acceptor residual anisotropies[5]:

$$r_c = \sqrt{r_{\mathrm{DD}}(t \to \infty)}\sqrt{r_{\mathrm{AA}}(t \to \infty)} \tag{5}$$

We determined the combined residual anisotropies based on the FRET-subpopulations ($r_{\mathrm{c}}^{\mathrm{a}}$), as well as based on the donor- and acceptor-only subpopulations ($r_{\mathrm{c}}^{\mathrm{b}}$). We decided to show both values because $r_{\mathrm{c}}^{\mathrm{a}}$ suffered from weak statistics. Although $r_{\mathrm{c}}^{\mathrm{b}}$ can be biased towards lower values due to remaining free dyes in solution, we believe that this is an important information, especially as the amount of free dye is very low. All values are shown in Supplementary Table 1.

## Protein modeling.

The yeast wild type Hsp90 dimer model was created by applying MODELLER[14] to one Hsp90 monomer (chain A) from the yeast Hsp90 crystal structure (PDB ID 2CG9)[15] to add missing loops and revert point mutations. The dimer was then reconstituted in vmd[16] by copying the full length monomer and aligning both monomers with the protein backbone of 2CG9. ATP was introduced into the binding site according to the coordinates in 2CG9. We need to point out that this crystal structure does contain ATP coordinates, but was actually crystallized using AMPPNP. For modeling structures with bound ADP and phosphate, we manually introduced a geometry change of the ATP $\gamma$-phosphate as it should appear in a $S_N2$ nucleophilic attack by a water molecule. The resulting ADP + Pi complex thus represents a structure immediately after the hydrolysis reaction and before any relaxation of the protein.

## MD simulations and data analysis.

All simulations of the Hsp90 dimer water were carried out using Gromacs 2016 (Ref. 17) using the Amber99SB*ILDN-parmbsc0-$\chi_{OL3}$ + AMBER99ATP/ADP force field,[18] which is an extension of AMBER99SB*ILDN[19–21] and contains improved parameters of ATP/ADP,[22,23] glycosidic torsions[24] and magnesium.[25] We modelled $P_i$ as $H_2PO_4^-$, as would be expected from the addition of a water molecule to $P_\gamma$. Missing $H_2PO_4^-$ and AMPPNP parameters were generated with antechamber[26] and acpype[27] using GAFF atomic parameters[28] and AM1/BCC charges[29] based on a protocol we have used before.[30] Minimum angles involving the N–H group between $P_\beta$ and $P_\gamma$ were derived from an AMPPNP structure minimised at the B3LYP/6-31G* level using Gaussian09.[31] The quality of AMPPNP simulation parameters was checked by comparison of a AMPPNP structure minimised in vacuo with the quantum mechanically minimised structure.

The simulation system consisted of a dodecahedral box of 17.5 nm side length filled with ca. 120,000 TIP3P water molecules.[32] Sodium and chloride ions were added to result in a charge neutral box with a 154 mM ion solution. We used a 2 fs time step and constrained hydrogen bonds by the LINCS algorithm.[33] Electrostatics were described by the particle mesh Ewald (PME) method.[34] Cutoffs were set to 1 nm for van der Waals interactions and a minimum of 1 nm for PME real space. Simulations were carried out in a NPT ensemble

with the temperature set to 310 K and the pressure to 1 bar. Temperature control was achieved by the Bussi velocity rescaling thermostat[35] (coupling time constant of 0.8 ps), and pressure control via the Parrinello-Rahman barostat[36] (isotropic pressure coupling, coupling time constant of 0.5 ps, compressibility of $4.5\text{x}10^{-5}$ $\text{bar}^{-1}$).

Simulation boxes were first minimised using the conjugate gradient method with position restraints on protein, nucleotide and phosphate heavy atoms. By starting with different initial velocity distributions, five statistically independent simulation replicas were calculated for each nucleotide load investigated. After a first 100 ps equilibration simulation with position restraints, each unbiased equilibrium production simulation was run for a total trajectory length of 1 µs. Simulations with modelled hydrolysis were subjected to a second equilibration run of 100 ps trajectory length with a step size of 0.2 fs and removed restraints to allow the binding site to adjust to the presence of the free phosphate molecule.

To access molecular details of the population shifts observed in smFRET, we performed MD simulations of the Hsp90 dimer with different nucleotides bound (Tab. S2). We checked if simulations resulted in a defined structural ensemble by assessing the time traces of the $C_\alpha$ root mean square displacement (RMSD) of the initial secondary structure elements within the full dimer (excluding the charged loop at sequence positions 208 to 280) and the radius of gyration of the full protein. As an example, Fig. S9 displays the structural development of the global protein in a representative ATP-bound simulation. For all simulations, the initial development away from the starting structure appears to be completed after 0.5 µs.

Interprotein distances were assessed using a correlation-based contact principal component analysis (conPCA)[37,38] on the last 0.5 µs of each simulation. We took into account all minimal interresidue distances $\mathbf{d} = \{d_{ij}\}$ within and between the N-domain and M-loops that lie within a 0.45 nm cutoff in the final structures after 1 µs of all 25 simulations. conPCA builds a correlation matrix

$$\sigma_{ij} = \langle d_i - \langle d_i \rangle \rangle \langle d_j - \langle d_j \rangle \rangle / \sigma_i \sigma_j, \tag{6}$$

with distance variances $\sigma_i$ and $\sigma_j$ which after diagonalisation yields $n$ eigenvectors $\mathbf{e}^{(\mathbf{n})}$ that are aligned with the maximal correlation within the data set, and $n$ eigenvalues $v^{(n)}$ that determine the contribution of eigenvector $n$ to the overall correlation. The principal

components $PC_n$ are then obtained via the projection

$$PC_n = \mathbf{e^{(n)}} \cdot \mathbf{d}. \tag{7}$$

Visualization of molecular data was performed with vmd.[16]

For nonequilibrium targeted molecular dynamics simulations,[39,40] we employed the PULL code from Gromacs. Prior to these simulations, we removed bulk solvent and ions from the final structure of one of the ADP+$P_i$ simulations after 1 µs simulation time, mimicking the influence of water by setting the relative permittivity $\epsilon_r = 78$ in the simulation, while lowering the overall friction of the system. For a simulation length of 1 ns, we pulled the $C_\beta$ atoms of the distance pairs 298-327 and 327-452 with a constant constraint velocity of 1 m/s, and the 298-452 pair with 2 m/s, respectively, which roughly corresponds to the changes in $R_{\langle E \rangle}$ observed between closed states A and B (see Fig. 4a, main text). We then use the apparent distances of $R_{\langle E \rangle}$ as read-out parameter, as they are derived from fluorophore accessible volume, and not the $C_\beta$ distance. Additionally, we did not manipulate 142-597, but use it as a control parameter.

The intention of the pulling simulations was to test if the beginning of the closure of the central folding substrate binding site observed in unbiased simulations is continued on large timescales, and if this finally yields the distance distributions of closed state B observed in experiments. We had to choose some lines along which we applied the pulling bias. As shown in Fig. 1, the connection lines of the 298-327, 298-452 and 327-452 distance pairs manipulated by our approach are crossing exactly this binding site. We therefore pulled along them to enforce the proteins closure. The control set 142-597 connecting axis goes right through the M domain, using it as a (linear) pulling coordinate is therefore not helpful to enforce folding binding site closure, which was the reason why we let it move freely as a control group.

**Comparing smFRET data to MD simulation data.**

Usually, FRET experiments and MD simulations are compared by converting dye distances into $C_\beta$-atom distances, using geometric arguments concerning linker lengths and flexibility. Since the latter usually involves ill-defined assumptions, we instead compared

measured and calculated $R_{\langle E \rangle}$. That is, we directly calculate the expected $R_{\langle E \rangle}$ for each simulations snapshot using the FPS software,[4] which analyses the volume a dye can access within the linker length around a given $C_\beta$-atom.[3,41] This yields the $R_{\langle E \rangle}$ distributions shown in Fig. 3a, which can be directly compared to the mean experimental distance $\mu$. The approach assumes isotropic averaging of dipoles during FRET, which can be verified via the low combined anisotropy of the FRET dyes (see Tab. S1 and Ref.[3]). Even in case of partial anisotropic averaging, this approach is preferential over simple $C_\beta$ estimation, as the volumes accessible to the fluorophores significantly depend on the structures appearing during the MD simulation.

## SUPPLEMENTARY TABLES

### smFRET distance measurements

Tab. S 1. Results from smFRET and anisotropy measurements. For each FRET pair ('donor position'-'acceptor position') and nucleotide condition Förster radii $R_0$, fractions of the closed states A and B, mean fluorophore distances $\mu$ of closed states A and B, uncertainties $\sigma$ and combined residual anisotropies determined from FRET populations $r_C^a$ and donor- and acceptor-only populations $r_C^b$ are summarised. Note, that for one distance in principle two smFRET experiments can be examined which is due to swapping of the donor- and acceptor-dye position. Swapping of donor- and acceptor-dyes can have a small effect on the Förster radius because of its environment-sensitivity. As seen from the table, we performed these measurements for some distances as a check for self-consistency. For details on the determination of the combined residual anisotropies, please refer to the SI Methods part C. For details on the error determination, please refer to the Methods section in the main text.

| FRET pair | Nucleotide | $\mathbf{R_0}$ [nm] | frac$_A$ | $\mu_\mathbf{A}$ [nm] | $\sigma_\mathbf{A}$ [nm] | frac$_B$ | $\mu_\mathbf{B}$ [nm] | $\sigma_\mathbf{B}$ [nm] | $r_\mathbf{c}^a$ | $r_\mathbf{c}^b$ |
|---|---|---|---|---|---|---|---|---|---|---|
| **142-597; 597-142** | apo | 5.41; 5.40 | 0.15 | 7.83 | 1.5E-3 | 0.09 | 5.67 | 0.02 | 0.20 | 0.20 |
| **142-597; 597-142** | ATP | 5.41; 5.40 | 0.15 | 7.83 | 0.09 | 0.15 | 5.67 | 0.72 | 0.21 | 0.17 |
| **597-142** | ADP | 5.40 | 0.15 | 7.83 | 0.62 | 0.22 | 5.67 | 0.18 | 0.20 | 0.15 |
| **142-597; 597-142** | AMPPNP | 5.41; 5.40 | 0.15 | 7.83 | 0.08 | 0.32 | 5.67 | 0.33 | 0.20 | 0.17 |
| **327-298** | apo | 6.37 | 0.23 | 5.22 | 0.70 | 0.03 | 4.52 | 0.41 | 0.24 | 0.17 |
| **327-298** | ATP | 6.37 | 0.23 | 5.22 | 1.27 | 0.03 | 4.52 | 0.01 | 0.24 | 0.17 |
| **327-298** | ADP | 6.37 | 0.18 | 5.22 | 0.64 | 0.00 | 4.52 | 2.61 | 0.21 | 0.15 |
| **327-298** | AMPPNP | 6.37 | 0.18 | 5.22 | 0.29 | 0.29 | 4.52 | 0.20 | 0.22 | 0.13 |
| **327-298** | ATP$\gamma$S | 6.37 | 0.17 | 5.22 | 0.29 | 0.15 | 4.52 | 0.01 | 0.21 | 0.12 |
| **298-452; 452-298** | apo | 6.31; 6.41 | 0.30 | 5.98 | 0.98 | 0.05 | 4.77 | 0.35 | 0.21 | 0.12 |
| **298-452; 452-298** | ATP | 6.31; 6.41 | 0.21 | 5.98 | 1.12 | 0.09 | 4.77 | 0.33 | 0.25 | 0.15 |
| **298-452; 452-298** | ADP | 6.31; 6.41 | 0.21 | 5.98 | 0.96 | 0.05 | 4.77 | 0.15 | 0.25 | 0.13 |
| **298-452; 452-298** | AMPPNP | 6.31; 6.41 | 0.26 | 5.98 | 0.59 | 0.13 | 4.77 | 0.30 | 0.22 | 0.12 |
| **298-452; 452-298** | ATP$\gamma$S | 6.31; 6.41 | 0.19 | 5.98 | 0.90 | 0.17 | 4.77 | 0.46 | 0.23 | 0.14 |
| **327-452** | apo | 6.32 | 0.00 | 5.91 | 1.14 | 0.06 | 4.80 | 0.23 | 0.19 | 0.15 |
| **327-452; 452-327** | ATP | 6.32; 6.47 | 0.07 | 5.91 | 1.15 | 0.08 | 4.80 | 0.13 | 0.24 | 0.15 |
| **327-452** | ADP | 6.32 | 0.15 | 5.91 | 0.75 | 0.14 | 4.80 | 0.16 | 0.20 | 0.09 |
| **327-452; 452-327** | AMPPNP | 6.32; 6.47 | 0.15 | 5.91 | 0.27 | 0.32 | 4.80 | 0.19 | 0.21 | 0.14 |
| **452-327** | ATP$\gamma$S | 6.47 | 0.08 | 5.91 | 0.84 | 0.15 | 4.80 | 0.37 | 0.20 | 0.11 |

## Simulation data

| protein | simulation No. | RMSD converged? | convergence time / ns | RMSD convergence | adenine bound? | Arg380 bound? |
|---|---|---|---|---|---|---|
| apo | 0 | yes | 100 | 4-5.5 | - | - |
| | 1 | yes | | | - | - |
| | 2 | yes | | | - | - |
| | 3 | yes | | | - | - |
| | 4 | yes | | | - | - |
| 2ATP | 0 | yes | 350 | 4,5 | ok | ok |
| | 1 | yes | 300 | 3,35 | ok | ok |
| | 2 | yes | 50 | 4.5 | ok | ok |
| | 3 | yes | 50 | 3.5 | B slightly moved | ok |
| | 4 | yes | 350 | 3.7 | ok | ok |
| ATP + ADP + $P_i$ | 0 | yes | 100 | 4.7 | jump (both) | ok |
| | 1 | yes | 200 | 4.6 | ok | ok |
| | 2 | yes | 300 | 4.3 | ok | ok |
| | 3 | yes | 250 | 5-5.5 | ok | no |
| | 4 | yes | 50 | 3.7 | jump/turn | no |
| 2ADP + $P_i$ | 0 | yes | 50 | 3-4.5 | ok | ok |
| | 1 | yes | 75 | 4 | ok | both unbind |
| | 2 | yes | 200 | 4 | ok | both unbind |
| | 3 | yes | 50 | 4-4.5 | ok | ok |
| | 4 | yes | 100 | 5-5.5 | ok | ok |
| 2MPPNP | 0 | yes | 250 | 4.5 | B jump | ok |
| | 1 | yes | 100 | 3.5 | ok | both unbind |
| | 2 | yes | 250 | 3.25 | ok | ok |
| | 3 | yes | 300 | 5.0 | B jump | ok |
| | 4 | yes | 300 | 4.0-5.0 | B jump | ok |

Tab. S 2. Statistics on simulation runs. Each simulation was carried out for 1 $\mu$s, except for 2ADP + $P_i$ / 1, which produced unreadable files after 0.784 $\mu$s due to a writing error.

# SUPPLEMENTARY FIGURES

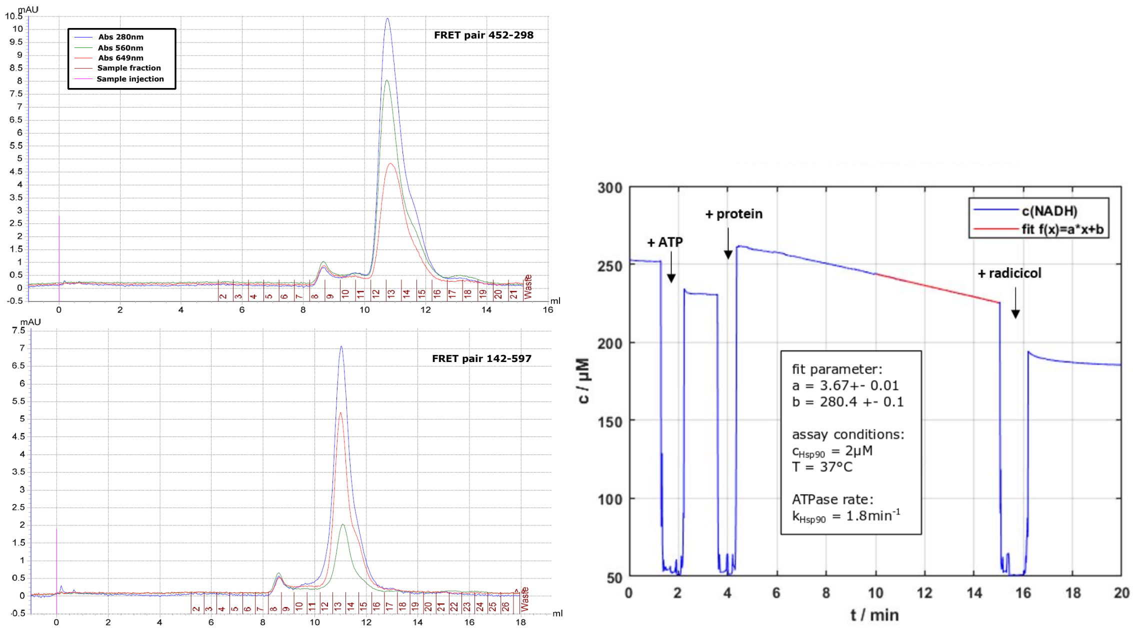


Fig. S 1. Left: SEC chromatograms for the FRET pairs 452-298 and 142-597 recorded on a Superdex 200 Increase 10/300 GL. In both cases, two main fractions are obtained with peaks at 8.3 and 13.0 ml, respectively. The first one is located within the exclusion volume of the column and therefore probably related to aggregates. We identify the second one as labelled Hsp90 dimers. The SEC profiles show, that amount of aggregates is very low with respect to the one of labelled Hsp90 dimers. Note, that in experiment, burst threshold and a stoichiometry filter further assure that aggregates are not involved in the data analysis. Right: ATPase activity test for the FRET pair 142-597 (all other FRET pairs have been tested and published before[5]). Hsp90 heterodimers were obtained by heating the respective homodimers, D142C and A597C, at 1:1 ratio for 43min at 43°C which promotes exchange of the monomers. Subsequent spin down (2h, 4°C, 16.9g) was performed to remove potential aggregates. The ATPase assay was performed at 37°C according to previous ATPase tests[42,43]. Absorbance at 340nm was monitored on a Lambda35 UV-VIS spectrometer (Perkin Elmer). 2 mM ATP was added to 0.2mM NADH, 10 u/ml lactate dehydrogenase, 6 u/ml pyruvate kinase and phosphoenolpyruvate solved in 40 mM HEPES, 150 mM KCl and 10 mM $MgCl_2$. The Hsp90 heterodimer 142-597 was added after the signal was stable. To determine the ATPase background, the reaction was stopped by radiciol (R2146-1MG, Sigma Aldrich) which specifically inhibits the ATPase activity of Hsp90. The ATP-turnover rate was determined from the slope of a linear fit to the decay of the signal after protein addition. We took the average from three tests and obtained $k_{Hsp90}$=(2.1±0.2) min$^{-1}$ which agrees well with previously determined values[5].

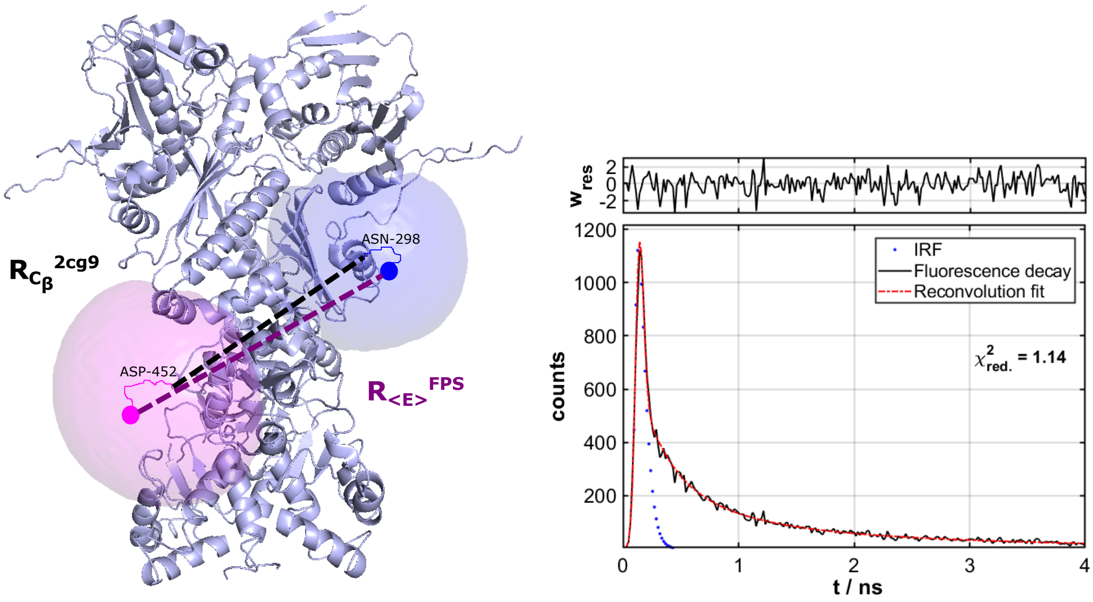


Fig. S 2. left: Estimation of the dye linker effect. In our study, distances obtained by smFRET and by MD simulation differ by the FRET linker which is attached to the $C_\beta$ atom of the respective Hsp90 amino acid. Here, the $C_\beta$-$C_\beta$ distance between position 452 and 298 is shown exemplarily for the 2CG9 structure (black dashed line). In order to account for the difference, for each structural MD snapshot the expected $R_{\langle E\rangle}^{\mathrm{FPS}}$ is calculated by the FPS Software.[4] Here, this is shown at the 2CG9 structure (purple line). Parameters used to calculate the accessible volumes of the dyes (blue and magenta spheres) were taken from Ref.[3]. Right: Subensemble lifetime analysis of the Hsp90 closed state B for the variant 452-298 with AMPPNP. $\tau_{\mathrm{D(A)}1}$=0.52±0.06 ns and $\tau_{\mathrm{D(A)}2}$=2.1±0.2 ns are obtained from a reconvolution approach with a biexponential model function. Fluorescence intensity of the donor channel after donor excitation is shown as black line, the reconvolution fit as red line and the instrument response function (IRF) in blue.

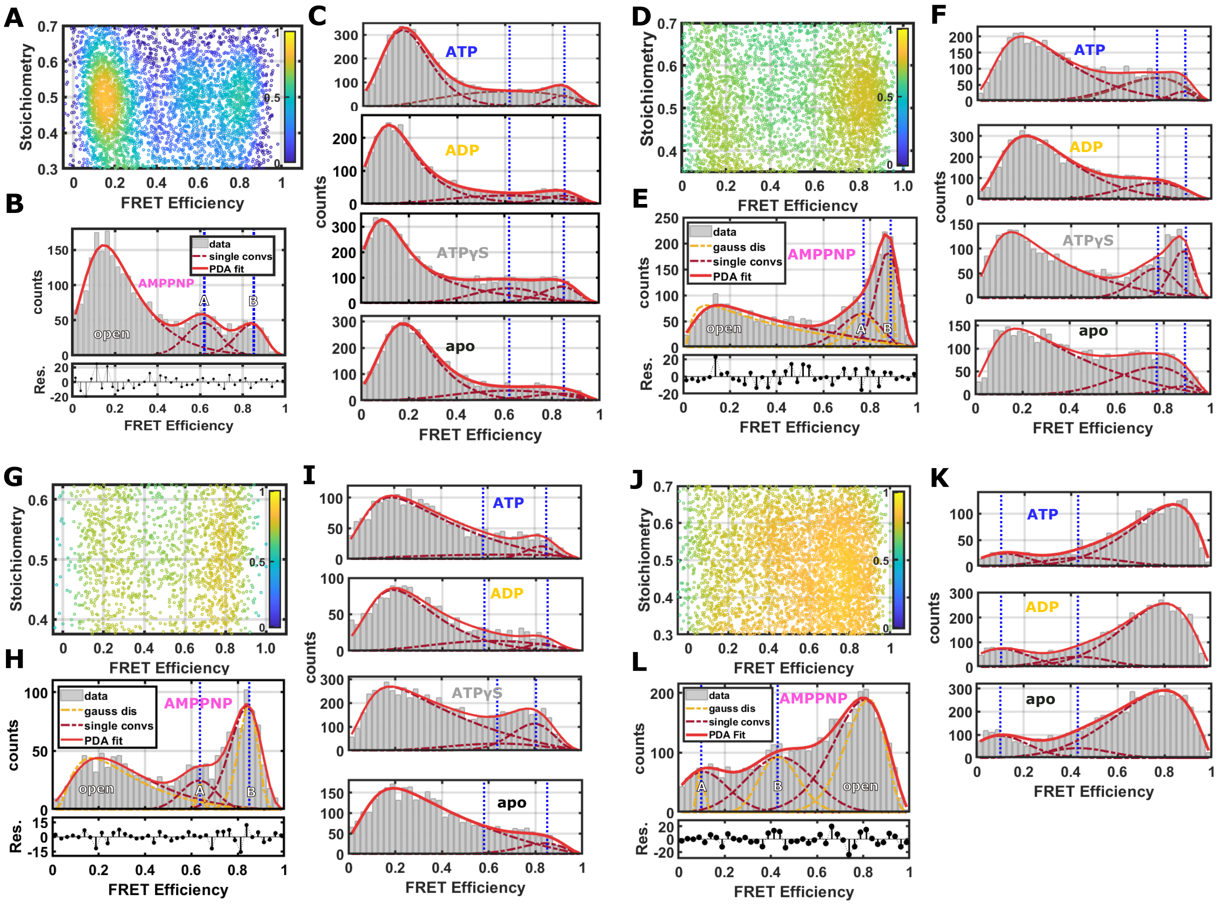

Fig. S 3. Summary of single-molecule FRET analysis for all four Hsp90 FRET pairs with different nucleotides. **a-c** distance 298-452, **d-f** distance 327-298, **g-h** distance 327-452 and **j-l** distance 142-597. **a, d, g, j** Corrected 2D FRET efficiency vs. stoichiometry histogram shown with AMPPNP for 298/452, 327/298, 327/452 and 597/142, respectively. Correction of FRET efficiencies and stoichiometries was performed as described in detail in Ref.[3]. For each pair three different FRET populations were identified. A kernel density estimator was used to visualise the burst density by colour. **b, e, h, l** Distances between the dyes and their distributions are extracted by Photon Distribution Analysis (PDA)[6,7]. Under AMPPNP conditions, the three Hsp90 states emerged the most clearly. Free three-state PDA fits are shown as red lines. Superposed single states are indicated by the dashed dark-red lines and shot-noise filtered states by the dashed orange lines. Distances of each population were extracted from the expectation values of each FRET state. These are indicated for the closed state A and B by vertical dashed blue lines. **c,f,i,k** PDA analysis shows the nucleotide dependence of the FRET pairs 298-452, 327-298, 327-452 and 142-597, respectively. Investigated nucleotide conditions are ATP, ADP, ATP$\gamma$S and apo. Fits were performed with FRET efficiencies fixed to the AMPPNP distances in order to investigate the nucleotide-specific state-population. Please note that for 327/452, we show experiments with donor and acceptor dye positions swapped (donor-acceptor positions for ATP$\gamma$S and AMPPNP are 452-327 and for ATP, ADP and apo conditions 327-452, respectively). Swapping leads to slight shifts of the mean FRET efficiencies (blue lines) between different subfigures because the different dye environments have small effects on the Förster radius and gamma factor. Considering the latter parameters, we arrive at the same distances for swapped dye pairs. In that manner we can exclude positions-specific photophysics-based uncertainties.

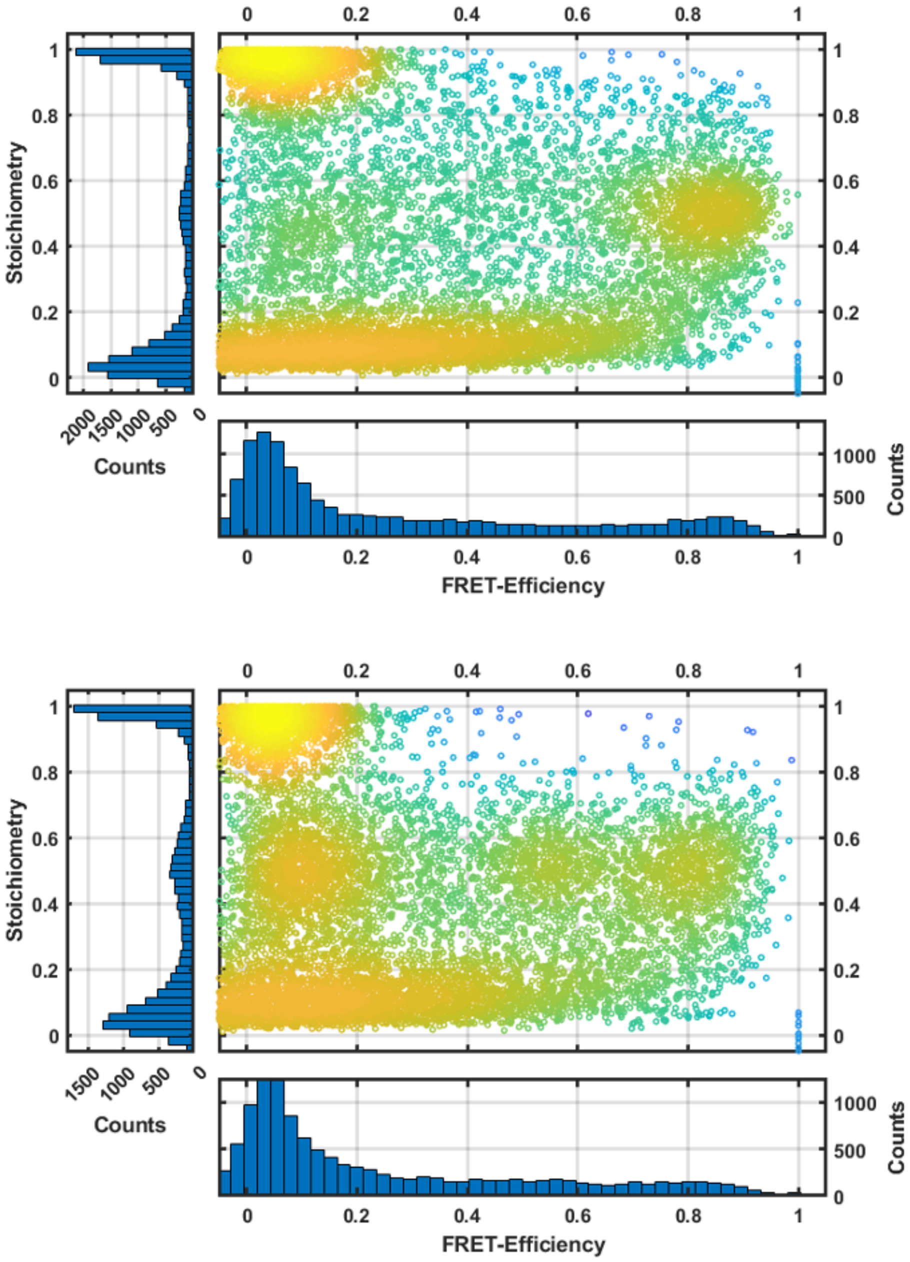


Fig. S 4. full E-S plot for the variant 327-298 (top) and as a comparison for the variant 452-298 (bottom). To indicate the burst rates we added histograms on the side of the E-S plot.

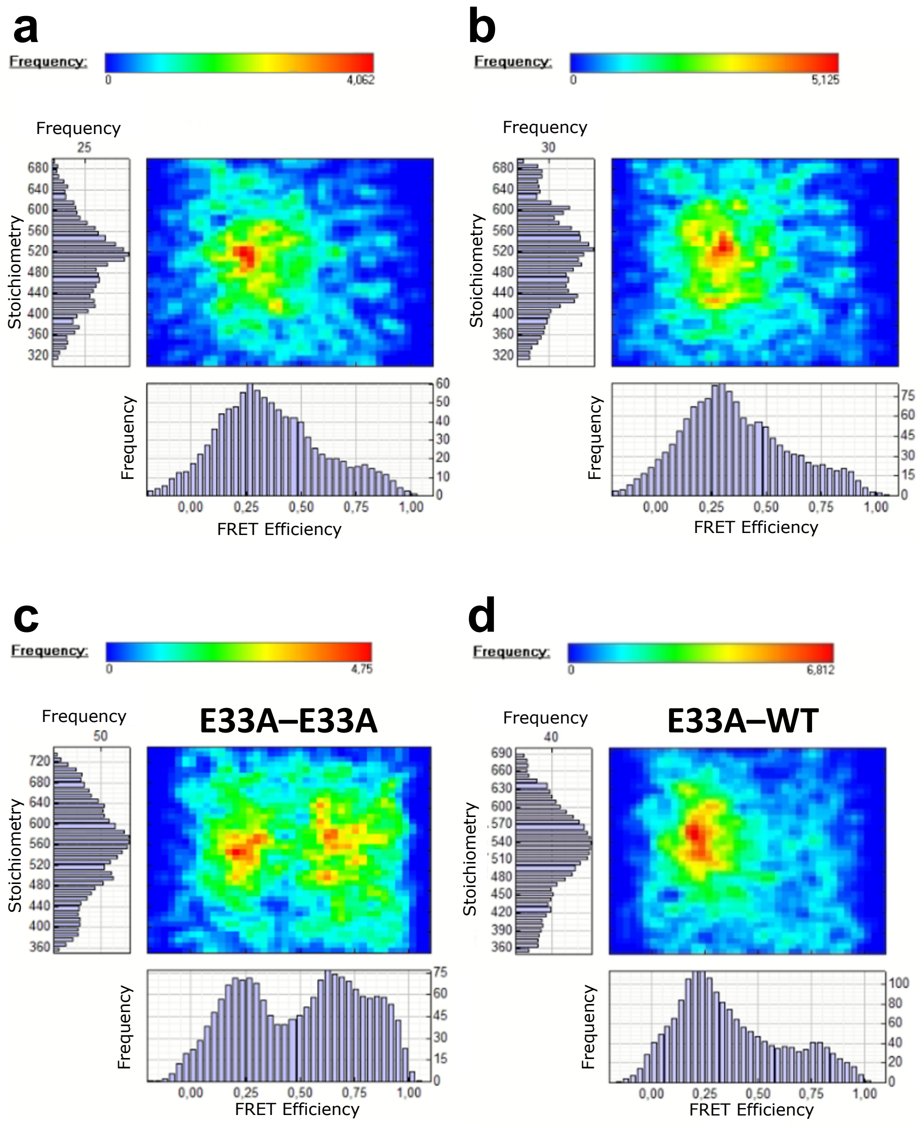


Fig. S 5. **a,b** ADP vs. ADP + Pi loaded Hsp90. **a** 1 mM ADP, **b** 1 mM ADP + 20 mM phosphate. Both conditions lead to near-identical FRET efficiency histograms. **c** E-S plot of the symmetric E33A-E33A variant, measured with 2mM ATP. The enrichment of the closed conformation in this mutant with impeded ATP hydrolysis[44–46] confirms that ATP stabilizes the closed state, and its hydrolysis is needed for the formation of the open state. **d** E-S plot of the asymmetric E33-WT dimer with 2mM ATP. In contrast to the symmetric E33A-E33A variant, Hsp90 is mainly found in the open state. The capability of Hsp90 to almost fully reopen is evidence for a single ATP hydrolysis to suffice for the reopening.

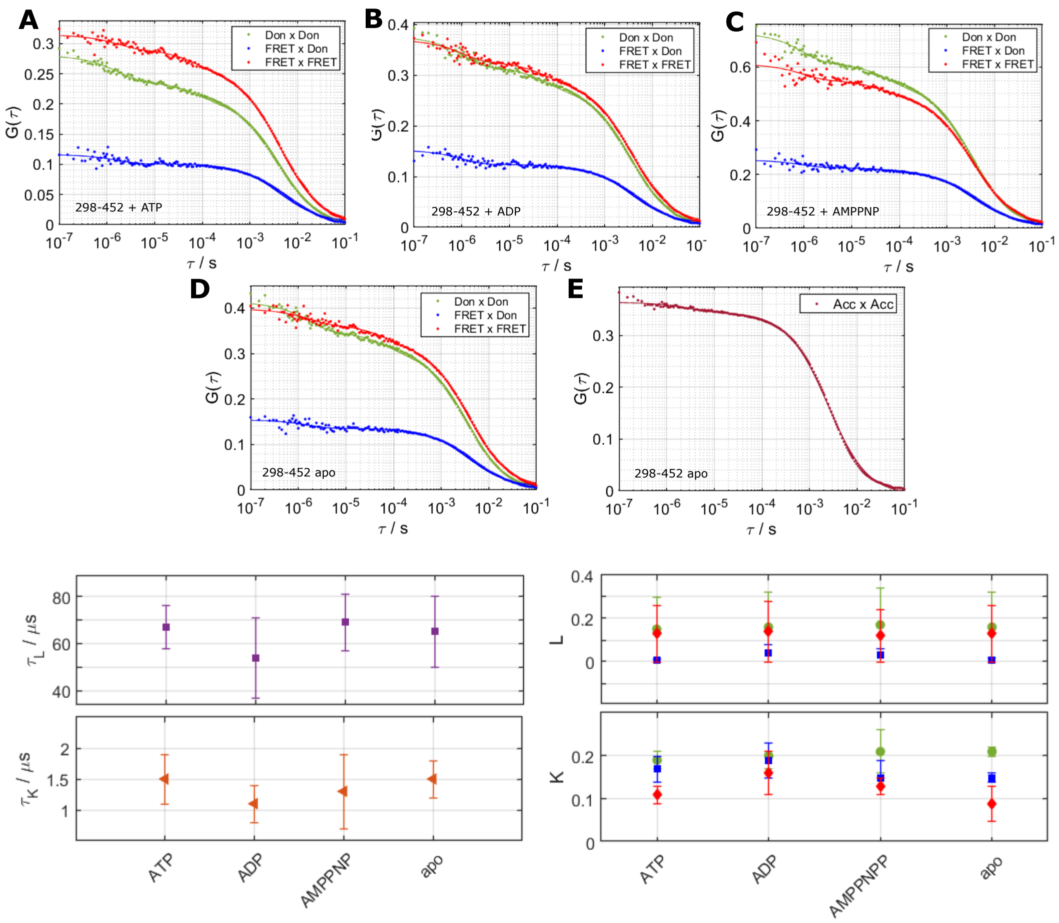


Fig. S 6. Top: FRET-FCS data and fits for FRET pair 298-452 of Hsp90. Auto-correlations DonxDon (green), FRETxFRET (red) and FRETxDon (blue) are shown for different nucleotide conditions: **a** ATP, **b** ADP, **c** AMPPNP and **d** apo. **e** Parallel and perpendicular parts of the acceptor signal after acceptor excitation are correlated for Hsp90 apo. The obtained AccxAcc correlation can be fit with model A, which includes simple 3D diffusion and a term for triplet kinetics only. The fact, that an additional kinetic term to fit AccxAcc is not necessary provides evidence, that we indeed see protein dynamics in a-d. Bottom: FRET-FCS fit results for FRET pair 298-452 of Hsp90. Shown are the relaxation times for the fast (orange) and the slow kinetic mode (purple), $\tau_K$ and $\tau_L$, respectively and the corresponding weights of the kinetic modes $K$ and $L$ for different nucleotide conditions. For each weight three data points are shown which are the weights obtained from the DonxDon correlation (green cycle), from the FRETxFRET correlation (red diamond) and from the FRETxDon correlation (blue square). We could not observe a significant trend with respect to the nucleotide present. See the paragraph "FRET-FCS analysis" on SI p. 6 for a more detailed discussion.

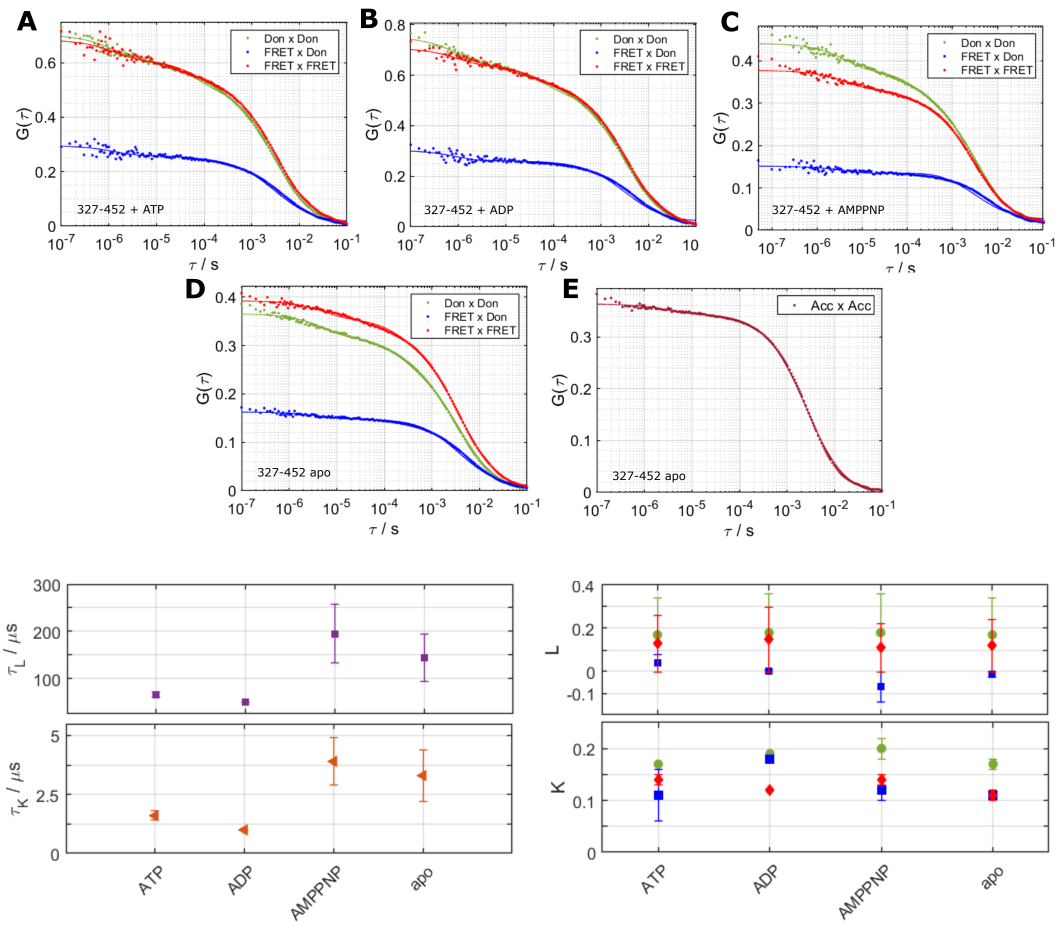


Fig. S 7. Top: FRET-FCS data and fits for FRET pair 327-452 of Hsp90. Auto-correlations DonxDon (green), FRETxFRET (red) and FRETxDon (blue) are shown for different nucleotide conditions: **a** ATP, **b** ADP, **c** AMPPNP and **d** apo. **e** Parallel and perpendicular parts of the acceptor signal after acceptor excitation are correlated for Hsp90 apo. The obtained AccxAcc correlation can be fit with model A, which includes simple 3D diffusion and a term for triplet kinetics only. The fact, that an additional kinetic term to fit AccxAcc is not necessary provides evidence, that we indeed see protein dynamics in a-d. Bottom: FRET-FCS fit results for FRET pair 327-452 of Hsp90. Shown are the relaxation times for the fast (orange) and the slow kinetic mode (purple), $\tau_K$ and $\tau_L$, respectively and the corresponding weights of the kinetic modes $K$ and $L$ for different nucleotide conditions. For each weight three data points are shown which are the weights obtained from the DonxDon correlation (green cycle), from the FRETxFRET correlation (red diamond) and from the FRETxDon correlation (blue square). As for the FRET pair shown before, we could not observe a significant trend with respect to the nucleotide present. See the paragraph "FRET-FCS analysis" on SI p. 6 for a more detailed discussion

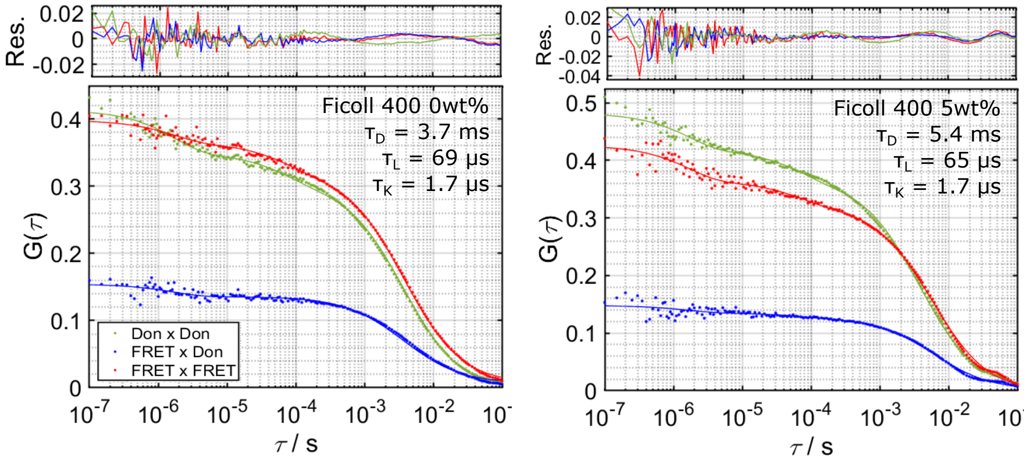


Fig. S 8. FRET-FCS data and fits for Hsp90 FRET pair 298-452 in standard solvent and solvent with increased viscosity due to the addition of 5wt% Ficoll 400. Upon increasing viscosity the diffusion rate $\tau_D$ increases, while timescales of the fast kinetic Hsp90 modes ($\tau_K$ and $\tau_L$, respectively) do not differ between both conditions. This rules out rotational diffusion as main cause of the fast timescales observed.

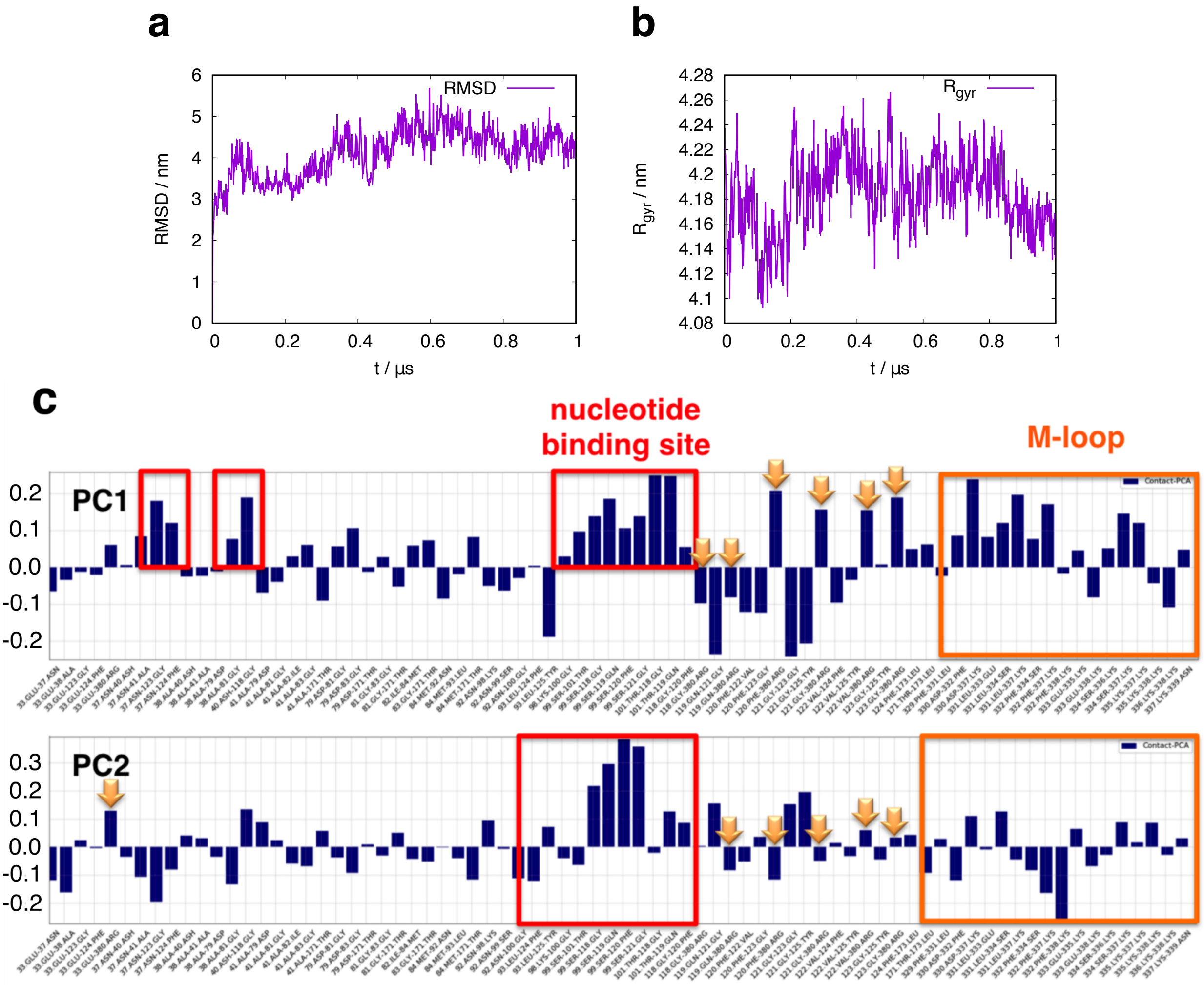


Fig. S 9. Time development of **a** secondary structure $C_\alpha$ root mean square displacement and **b** global radius of gyration in a representative trajectory with ATP-bound protein. **c** content of individual contacts within principal components 1 and 2. Nucleotide binding site contacts in red, M loop contacts in orange. Contacts of Arg380 highlighted with yellow arrows.

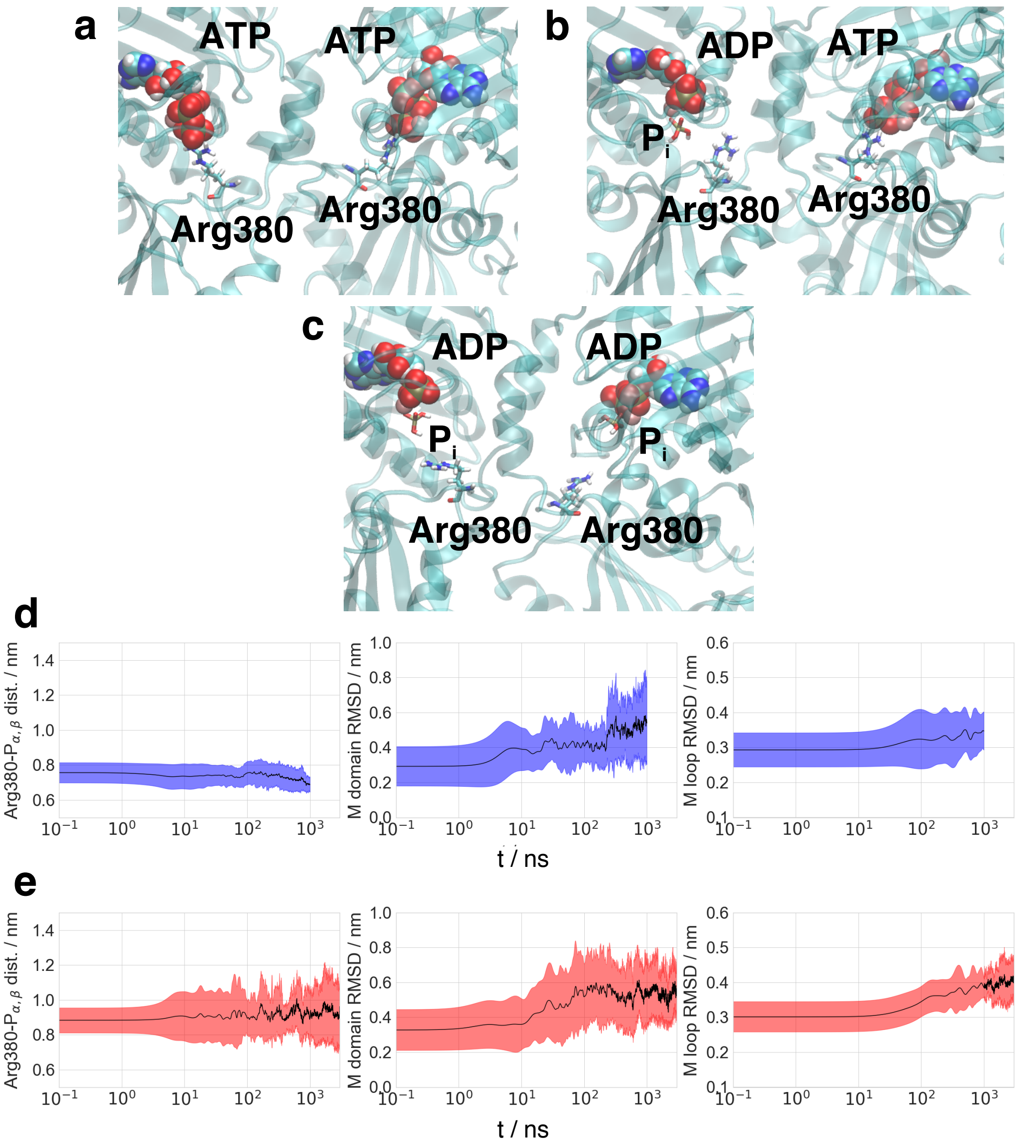


Fig. S 10. **a-c** changes of the binding site and Arg380 between ATP-bound, single hydrolysis and doubly hydrolyzed states. ATP, ADP and magnesium ion as van der Waals spheres, $P_i$ and Arg380 as sticks. Displayed are the respective structures at the end of one selected simulation for all three states (simulations "1" in Tab. S2). **d,e** Time-resolved average structural changes in **d** ATP and **e** ATP/ADP state simulations, respectively. Mean values displayed in black lines, standard deviations as coloured traces. Displayed observables are: distance between the Arg380 CZ atom (center atom of guanidyl group) and the respective nucleotide $P_\alpha$ / $P_\beta$ mass center; RMSD of the C$\alpha$ atoms from secondary structure elements (helices, sheets) in the M-domain after fit of the respective N-domain C$\alpha$ atoms from secondary structure elements (helices, sheets) - measures the shift in position of the M-domain in respect to the N-domain; RMSD of the two internal M loops in respect to the conformation of the substrate bound cryo-EM structure (PDB ID 5FWK[47]).

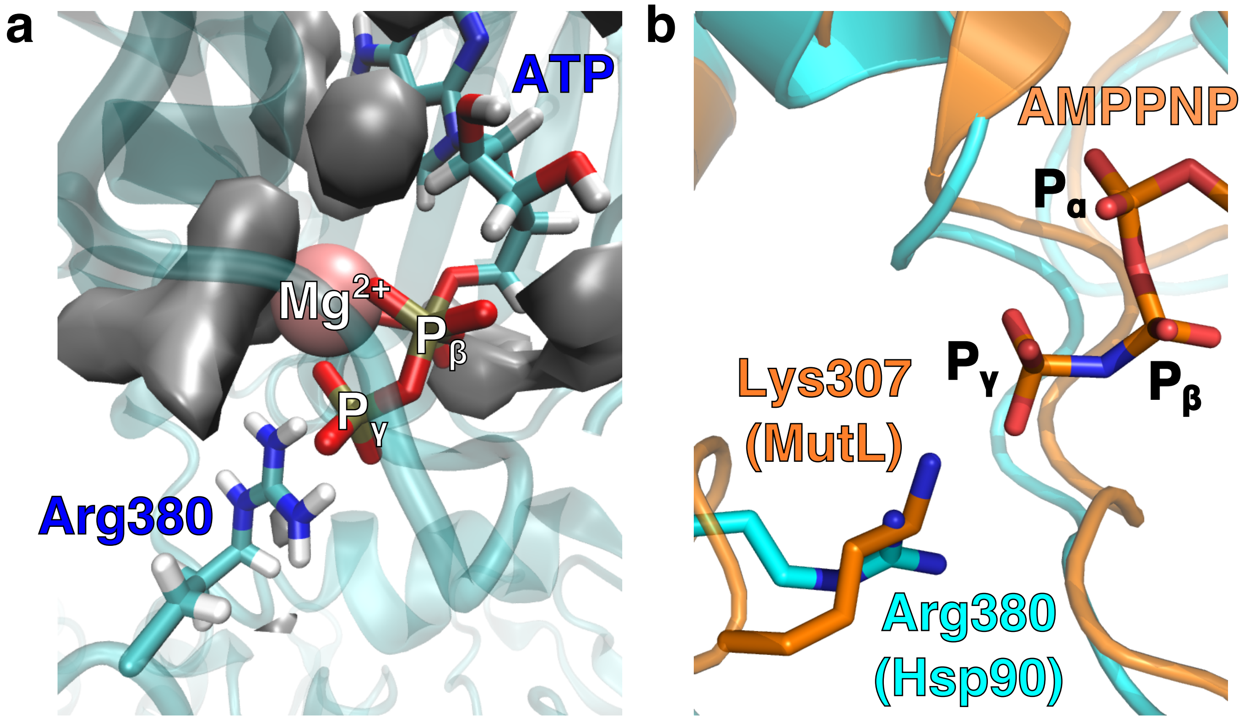


Fig. S 11. **a** structurally relevant water positions in ATP-bound Hsp90 around the nucleotide. Protein structure as transparent cartoon, Arg380 and ATP as sticks, magnesium ion as van der Waals sphere. Volumes with a minimal residence probability $P \geq 0.4$ for a water molecule to be found within 500–1000 ns of free MD simulation (”water densities”)[48] as grey isosurfaces. **b** Comparison of position of Arg380 in Hsp90 (PDB ID 2CG9)[15] and Lys307 in MutL (PDB ID 1B63)[49].

## SUPPLEMENTARY REFERENCES


[1]M. Götz, P. Wortmann, S. Schmid and T. Hugel, *Methods Enzymol.*, Elsevier, 2016, vol. 581, pp. 487–516.

[2]M. Mickler, M. Hessling, C. Ratzke, J. Buchner and T. Hugel, *Nat. Struct. Mol. Biol.*, 2009, **16**, 281.

[3]B. Hellenkamp, S. Schmid, O. Doroshenko, O. Opanasyuk, R. Kühnemuth, S. R. Adariani, B. Ambrose, M. Aznauryan, A. Barth, V. Birkedal *et al.*, *Nat. Methods*, 2018, **15**, 669.

[4]S. Kalinin, T. Peulen, S. Sindbert, P. J. Rothwell, S. Berger, T. Restle, R. S. Goody, H. Gohlke and C. A. Seidel, *Nat. Methods*, 2012, **9**, 1218.

[5]B. Hellenkamp, P. Wortmann, F. Kandzia, M. Zacharias and T. Hugel, *Nat. Methods*, 2017, **14**, 174–180.

[6]M. Antonik, S. Felekyan, A. Gaiduk and C. A. M. Seidel, *J. Phys. Chem. B*, 2006, **110**, 6970–6978.

[7]E. Nir, X. Michalet, K. M. Hamadani, T. A. Laurence, D. Neuhauser, Y. Kovchegov and S. Weiss, *J. Phys. Chem. B*, 2006, **110**, 22103–22124.

[8]W. Schrimpf, A. Barth, J. Hendrix and D. C. Lamb, *Biophys. J.*, 2018, **114**, 1518–1528.

[9]S. Kalinin, A. Valeri, M. Antonik, S. Felekyan and C. A. M. Seidel, *J. Phys. Chem. B*, 2010, **114**, 7983–7995.

[10]I. V. Gopich and A. Szabo, *Proc. Natl. Acad. Sci. USA*, 2012, **109**, 7747–7752.

[11]T. Torres and M. Levitus, *J. Phys. Chem. B*, 2007, **111**, 7392–7400.

[12]A. Barth, J. Hendrix, D. Fried, Y. Barak, E. A. Bayer and D. C. Lamb, *Proc. Natl. Acad. Sci. USA*, 2018, **115**, E11274–E11283.

[13]N. Vandenbekr, a. Barth, D. Borrenberghs, J. Hofkens and J. Hendrix, *J. Phys. Chem. B*, 2018, **122**, 4249–4266.

[14]B. Webb and A. Sali, *Curr. Protoc. Bioinformatics*, 2014, **47**, 5.6.1–5.6.32.

[15]M. M. U. Ali, S. M. Roe, C. K. Vaughan, P. Meyer, B. Panaretou, P. W. Piper, C. Prodromou and L. H. Pearl, *Nature*, 2006, **440**, 1013–1017.

[16]W. Humphrey, A. Dalke and K. Schulten, *J. Mol. Graph.*, 1996, **14**, 33–38.

[17]M. J. Abraham, T. Murtola, R. Schulz, S. Páll, J. C. Smith, B. Hess and E. Lindahl, *SoftwareX*, 2015, **1**, 19–25.

[18]A. Pérez-Villa, M. Darvas and G. Bussi, *Nucleic Acids Res.*, 2015, **43**, 8725–8734.

[19]V. Hornak, R. Abel, A. Okur, B. Strockbine, A. Roitberg and C. Simmerling, *Proteins*, 2006, **65**, 712–725.

[20]R. B. Best and G. Hummer, *J. Phys. Chem. B*, 2009, **113**, 9004–9015.

[21]K. Lindorff Larsen, S. Piana, K. Palmo, P. Maragakis, J. L. Klepeis, R. O. Dror and D. E. Shaw, *Proteins*, 2010, **78**, 1950–1958.

[22]K. L. Meagher, L. T. Redman and H. A. Carlson, *J. Comput. Chem.*, 2003, **24**, 1016–1025.

[23]A. Pérez, I. Marchán, D. Svozil, J. Sponer, T. E. Cheatham, C. A. Laughton and M. Orozco, *Biophys. J.*, 2007, **92**, 3817–3829.

[24]M. Zgarbová, M. Otyepka, J. Sponer, A. Mládek, P. Banáš, T. E. Cheatham and P. Jurečka, *J. Chem. Theory Comput.*, 2011, **7**, 2886–2902.

[25]O. Allnér, L. Nilsson and A. Villa, *J. Chem. Theory Comput.*, 2012, **8**, 1493–1502.

[26]J. Wang, W. Wang, P. A. Kollman and D. A. Case, *J. Mol. Graph. Model.*, 2006, **25**, 247–260.

[27]A. W. Sousa da Silva and W. F. Vranken, *BMC Res. Notes*, 2012, **5**, 367.

[28]J. M. Wang, R. M. Wolf, J. W. Caldwell, P. A. Kollman and D. A. Case, *J. Comput. Chem.*, 2004, **25**, 1157–1174.

[29]A. Jakalian, B. L. Bush, D. B. Jack and C. I. Bayly, *J. Comput. Chem.*, 2000, **21**, 132–146.

[30]C. Zanobini, O. Bozovic, B. Jankovic, K. L. Koziol, P. J. M. Johnson, P. Hamm, A. Gulzar, S. Wolf and G. Stock, *J. Phys. Chem. B*, 2018, **122**, 10118–10125.

[31]M. J. Frisch, G. W. Trucks, H. B. Schlegel, G. E. Scuseria, M. A. Robb, J. R. Cheeseman, G. Scalmani, V. Barone, B. Mennucci, G. A. Petersson, H. Nakatsuji, M. Caricato, X. Li, H. P. Hratchian, A. F. Izmaylov, J. Bloino, G. Zheng, J. L. Sonnenberg, M. Hada, M. Ehara, K. Toyota, R. Fukuda, J. Hasegawa, M. Ishida, T. Nakajima, Y. Honda, O. Kitao, H. Nakai, T. Vreven, J. A. Montgomery, Jr, J. E. Peralta, F. Ogliaro, M. Bearpark, J. J. Heyd, E. Brothers, K. N. Kudin, V. N. Staroverov, R. Kobayashi, J. Normand, K. Raghavachari, A. Rendell, J. C. Burant, S. S. Iyengar, J. Tomasi, M. Cossi, N. Rega, J. M. Millam, M. Klene, J. E. Knox, J. B. Cross, V. Bakken, C. Adamo, J. Jaramillo, R. Gomperts, R. E. Stratmann, O. Yazyev, A. J. Austin, R. Cammi, C. Pomelli, J. W. Ochterski, R. L. Martin, K. Morokuma, V. G. Zakrzewski, G. A. Voth, P. Salvador, J. J. Dannenberg, S. Dapprich, A. D. Daniels, Ö. Farkas, J. B. Foresman, J. V. Ortiz, J. Cioslowski and D. J. Fox, *Gaussian Inc*, 2009.

[32]W. L. Jorgensen, J. Chandrasekhar, J. D. Madura, R. W. Impey and M. Klein, *J. Chem. Phys.*, 1983, **79**, 926.

[33]B. Hess, H. Bekker, H. J. C. Berendsen and J. G. E. M. Fraaije, *J. Comput. Chem.*, 1997, **18**, 1463–1472.

[34]T. Darden, D. York and L. Pedersen, *J. Chem. Phys.*, 1993, **98**, 10089–10092.

[35]G. Bussi, D. Donadio and M. Parrinello, *J. Chem. Phys.*, 2007, **126**, 0141011.

[36]M. Parrinello and A. Rahman, *J. Appl. Phys.*, 1981, **52**, 7182–7190.

[37]M. Ernst, F. Sittel and G. Stock, *J. Chem. Phys.*, 2015, **143**, 244114.

[38]M. Ernst, S. Wolf and G. Stock, *J. Chem. Theory Comput.*, 2017, **13**, 5076–5088.

[39]J. Schlitter, M. Engels and P. Krüger, *J. Mol. Graph.*, 1994, **12**, 84–89.

[40]S. Wolf, M. Amaral, M. Lowinski, F. Vallée, D. Musil, J. Güldenhaupt, M. K. Dreyer, J. Bomke, M. Frech, J. Schlitter and K. Gerwert, *J. Chem. Inf. Model.*, 2019, **59**, 5135–5147.

[41]S. Sindbert, S. Kalinin, H. Nguyen, A. Kienzler, L. Clima, W. Bannwarth, B. Appel, S. Müller and C. A. M. Seidel, *J. Am. Chem. Soc.*, 2011, **133**, 2463–2480.

[42]J. A. Ali, A. P. Jackson, A. J. Howells and A. Maxwell, *Biochemistry*, 1993, **32**, 2717–2724.

[43]M. Hessling, K. Richter and J. Buchner, *Nat. Struct. Mol. Biol.*, 2009, **16**, 287–293.

[44]B. Panaretou, C. Prodromou, S. M. Roe, R. O'Brien, J. E. Ladbury, P. W. Piper and L. H. Pearl, *EMBO J.*, 1998, **17**, 4829–4836.

[45]W. M. J. Obermann, H. Sondermann, A. A. Russo, N. P. Pavletich and F. U. Hartl, *J. Cell Biol.*, 1998, **143**, 901–910.

[46]S. M. Roe, M. M. U. Ali, P. Meyer, C. K. Vaughan, B. Panaretou, P. W. Piper, C. Prodromou and L. H. Pearl, *Cell*, 2014, **116**, 87–98.

[47]K. A. Verba, R. Y.-R. Wang, A. Arakawa, Y. Liu, M. Shirouzu, S. Yokoyama and D. A. Agard, *Science*, 2016, **352**, 1542–1547.

[48]S. Wolf, E. Freier and K. Gerwert, *ChemPhysChem*, 2008, **9**, 2772–2778.

[49]C. Ban, M. Junop and W. Yang, *Cell*, 1999, **97**, 85–97.
